# Outcomes of Different Regimens of Rivaroxaban and Aspirin in Cardiovascular Diseases: A Network Meta-Analysis

**DOI:** 10.3390/jcm14103437

**Published:** 2025-05-14

**Authors:** Mohammed Maan Al-Salihi, Adnan I. Qureshi

**Affiliations:** 1 Zeenat Qureshi Stroke Institute, University of Missouri, Columbia, MO 65211, USA; 2 Department of Neurology, University of Missouri, Columbia, MO 65211, USA

**Keywords:** rivaroxaban, aspirin, cardiovascular disease, coronary artery disease, network meta-analysis

## Abstract

**Background/Objectives:** Rivaroxaban is widely used to prevent thrombotic events in cardiovascular diseases (CVD). While various doses and combinations with aspirin have been evaluated across CVD subtypes, the optimal regimen remains unclear. This network meta-analysis aims to identify the most effective and safe rivaroxaban regimens, with or without aspirin, for patients with CVD. **Methods**: A systematic search of PubMed, Scopus, Cochrane Library, and Web of Science identified randomized-controlled trials (RCTs) assessing rivaroxaban, with or without aspirin, in CVD. Key outcomes included thromboembolic, hemorrhagic, and mortality events. A frequentist network meta-analysis (MetaInsight tool) was performed, using aspirin monotherapy as the reference. Subgroup analyses for coronary artery disease (CAD) were conducted. **Results**: Seven RCTs were included. Rivaroxaban 2.5 mg twice daily (“bis in die” (BID)) with aspirin showed the most significant venous thromboembolism (VTE) prevention (RR = 0.61, 95% CI [0.43–0.86]) but had the highest major bleeding risk (RR = 1.58, 95% CI [1.26–2]). Rivaroxaban 5 mg BID with aspirin showed the lowest myocardial infarction risk (RR = 0.78). Higher doses (20 mg BID) with aspirin were associated with an increased fatal bleeding risk (RR = 7.14, 95% CI [2.83–17.98]). Rivaroxaban 5 mg BID monotherapy had the highest hemorrhagic stroke risk (RR = 2.7, 95% CI [1.31–5.58]). In CAD, rivaroxaban 2.5 mg BID plus aspirin offered the lowest all-cause mortality (RR = 0.76, 95% CI [0.63–0.93]). **Conclusions**: Rivaroxaban 2.5 mg BID plus aspirin reduces VTE and lowers mortality in CAD but carries higher bleeding risks. Optimal regimen selection requires a careful risk–benefit balance.

## 1. Introduction

Cardiovascular diseases (CVDs) represent a major cause of morbidity and mortality worldwide, including a wide range of conditions, collectively categorized as CAD, cerebrovascular disease, peripheral artery disease (PAD), and aortic diseases [1]. Venous thromboembolism (VTE) is known as the third most common form of CVD, after CAD and cerebrovascular diseases [2]. All of these conditions, together with other cardiac pathologies, including atrial fibrillation and prosthetic valves, are associated with an increased risk of thrombosis and embolism, underscoring the importance of advocating for antithrombotic measures for both primary and secondary prophylaxis [3,4,5,6].

Aspirin has long been the key element of antiplatelet therapy for preventing cardiovascular events, yet its limited efficacy in reducing thromboembolism highlights the need for more effective treatment strategies [7]. A combination with anticoagulants like warfarin has been investigated, yielding superiority in efficacy over aspirin monotherapy, but was coupled with a significant risk of hemorrhage [8]. Rivaroxaban, a direct oral anticoagulant (DOAC), has emerged as a potential addition, or sometimes an alternative to antiplatelet therapy, due to its ability to inhibit thrombin synthesis by competitive inhibition of factor Xa, addressing a key pathway in thrombus formation [9]. It has gained approval for several conditions. Recently, rivaroxaban received approval from the Food and Drug Administration (FDA) for secondary prevention after acute myocardial infarction (MI) and PAD, adjunctive to dual antiplatelet therapy with aspirin and clopidogrel [10]. Choosing anticoagulants over antiplatelet agents or vice versa is primarily based on the underlying pathophysiology of thrombus formation in the specific condition and the associated risks of thromboembolic events.

Recent trials have explored the difference in efficacy and safety outcomes among different regimens of rivaroxaban with and without aspirin across several categories of CVD. There is evidence of an increase in bleeding risk with rivaroxaban in a dose-dependent manner [11,12]. Rivaroxaban 2.5 mg BID plus aspirin has been reported to have more favorable efficacy when compared to aspirin alone in stable CAD and PAD. However, this regimen still had more odds of bleeding events [13]. Other trials have reported mixed results, with some showing non-significant differences and even less favorable outcomes when using rivaroxaban in other clinical contexts [14,15]. This network meta-analysis seeks to address this gap by pooling evidence from available randomized controlled trials (RCTs). Herein, we compared the outcomes of different studied regimens of rivaroxaban with or without aspirin in different categories of CVD. This study aims to inform clinical decision-making and potentially guide updates to antithrombotic treatment guidelines.

Despite the growing body of research investigating rivaroxaban and its combination with aspirin in various cardiovascular conditions, there remains significant ambiguity regarding the optimal regimen for balancing efficacy and safety. Current studies have presented conflicting results, with some demonstrating substantial benefits in reducing thromboembolic events, while others have reported an elevated bleeding risks, limiting their generalizability to broader patient populations. To date, no network meta-analysis has comprehensively synthesized these data across diverse cardiovascular disease categories. This study, therefore, aims to address this critical gap in the literature by providing a systematic comparison of all available rivaroxaban regimens, both as monotherapy and in combination with aspirin. By pooling direct and indirect evidence, this study offers a unique opportunity to guide clinical decision-making and inform future updates to antithrombotic treatment guidelines.

## 2. Methods

The literature search and all steps for this network meta-analysis were conducted based on the guidelines established by the Preferred Reporting Items for Systematic Reviews and Meta-Analyses (PRISMA) checklist [16]. This systematic review has not been registered.

### 2.1. Search Strategy and Study Selection

We searched through 4 databases: PubMed, Scopus, Cochrane Library, and Web of Science (WOS), from their inception to 12 October 2024. The following terms were used: (“Acetylsalicylic Acid” OR Easprin OR Zorprin OR Colfarit OR Ecotrin OR “ASA” OR Acenterine OR Acetosal OR Acetosalin OR Ascoden-30 OR Aspergum OR Aspirin OR Aspirine OR Asteric OR Easprin OR Ecotrin OR Empirin OR “Nu-seals” OR Premaspin OR Rhodine OR Rhonal OR Tasprin) AND (Rivaroxaban OR Xarelto OR “Apo-rivaroxaban” OR “Mint-rivaroxaban” OR “Rivaroxaban Mylan” OR “Teva-rivaroxaban” OR “Pmsc-rivaroxaban” OR “Rivaroxaban Accord” OR “Sandoz Rivaroxaban” OR “Riva Rivaroxaban” OR “Ach-rivaroxaban” OR “Nra-rivaroxaban” OR “Reddy-rivaroxaban” OR “Taro-rivaroxaban” OR “PMS-rivaroxaban” OR “Pro-rivaroxaban” OR rivaroxabanum) AND (“Coronary Artery Diseases” OR “Stable cardiovascular diseases” OR “Artery Disease, Coronary” OR “Artery Diseases, Coronary “ OR “Coronary Arteriosclerosis” OR “ Coronary Arterioscleroses” OR “Arteriosclerosis, Coronary” OR “Atherosclerosis, Coronary” OR “Atheroscleroses, Coronary” OR ”Left Main Coronary Disease” OR “Left Main Disease” OR “Arterioscleroses, Coronary” OR “Left Main Diseases” OR “ischemic heart disease” OR ”Coronary Occlusion” OR “myocardial ischemia heart disease” OR “Stable angina” OR “Peripheral arterial diseases “ OR “Arterial occlusive disease”). The records were exported to Endnote for duplicate detection and screening. Two independent authors carried out the process of title and abstract screening, then the relevant records were subjected to further detailed full-text screening. In case of disagreements, a consensus with a third reviewer was taken into consideration.

### 2.2. Eligibility Criteria

A network meta-analysis was employed to evaluate the outcomes of multiple interventions through both direct and indirect comparisons within a network of studies. Studies were included if: (i) had rivaroxaban or aspirin in each study arm, (ii) were RCTs, and (iii) had data regarding comparative safety and efficacy outcomes. No restrictions were imposed in terms of the type of cardiovascular disease; all relevant studies were considered for inclusion to ensure a comprehensive evaluation of safety and efficacy outcomes whatever the type of CVD. This includes CAD, PAD, AF, or VTE. Our inclusion was restricted to studies published in English only. We excluded observational studies, conference proceedings, or abstracts without the full texts, and studies with arms different to our pre-specified criteria. No restrictions were applied based on cardiovascular disease subtype or doses of treatments.

### 2.3. Data Extraction and Quality Assessment

Details about the study, including the first author’s name, publication year, protocol (NCT) number, disease definition, intervention and control regimens, follow-up duration, and primary outcomes, were extracted and tabulated in a summary table. Baseline data, such as sample size in each study arm, age, gender distribution, comorbidities, and risk profile, were also extracted and presented in a separate table. The odds of different outcomes were extracted in a customized spreadsheet as events per total, along with details of each study (regimens and doses) to further define the different arms of the network. Quality assessment was performed using the Cochrane Risk of Bias 2 (RoB-2) tool [17].

### 2.4. Outcomes and Analysis

A frequentist network meta-analysis was performed to compare safety outcomes among the different network arms: hemorrhagic stroke, fatal bleeding, major bleeding as defined by Thrombolysis in Myocardial Infarction (TIMI) classification and the International Society on Thrombosis and Haemostasias (ISTH), ischemic stroke, MI, VTE, systemic embolism, death from cardiovascular causes, and death from any cause. Death from any cause equals death from cardiovascular causes plus non-cardiovascular causes. VTE includes deep vein thrombosis (DVT) plus pulmonary embolism. All analyses were performed using MetaInsight (University of Leicester, Leicester, UK), a web-based interface built on R (version 4.2.2, R Foundation for Statistical Computing, Vienna, Austria), Shiny (version 1.7.4, Posit Software, Boston, MA, USA), and the netmeta package (version 1.5-1, Freiburg, Germany) [18]. Since all outcomes of interest were dichotomous, risk ratios (RRs) with 95% confidence intervals (CIs) were used as the effect estimates. Intervals not including one was considered to be significant. We employed a frequentist random-effects model for direct and indirect comparisons. From the included studies, we defined 8 arms as follows: (aspirin + rivaroxaban 2.5 mg BID), (aspirin + rivaroxaban 10 mg OD), (aspirin and rivaroxaban 5 mg BID), (rivaroxaban 5 mg BID), (aspirin alone), (aspirin + thienopyridine), (rivaroxaban 10 mg OD), and aspirin and rivaroxaban 20 mg BID). A separate analysis was conducted in the studies focusing on CAD. Aspirin alone was used as the reference comparator in all analyses. Forest plots were generated to demonstrate the results obtained from the comparisons. Treatments were ranked in league tables, showing pairwise comparisons between different regimens.

## 3. Results

### 3.1. Search Results

**Figure 1:**
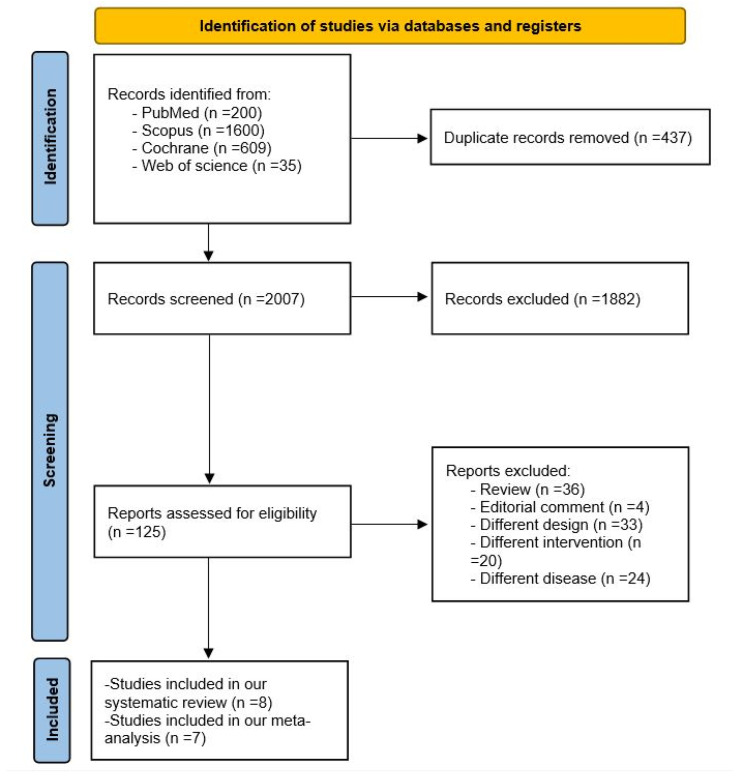
PRISMA flowchart.

Our search yielded 2444 records. After removing duplicates, a total of 2007 records were subjected to screening. After title and abstract screening, 1882 articles were excluded. A total of 125 studies were subjected to full-text screening. Finally, eight studies were included in this systematic review with one of them excluded from the analysis [11,12,13,14,15,19,20,21]. The PRISMA flowchart is demonstrated in Figure 1.

### 3.2. Study Characteristics and Narrative Synthesis

Direct and indirect evidence was synthesized through a network of seven RCTs, with a total of 61,944 cases [11,12,13,14,15,19,20]. The separate analysis on cardiovascular diseases included only four studies [11,12,13,14]. All studies were multinational except for the studies of Yasuda et al. and Maximiliano et al. which were located in Japan and Mexico, respectively [19,21]. Follow up durations varied with a range from 3 to 31 months. Table 1 and Table 2 summarize the characteristics and baseline data of the included studies.

The intervention and control arms across the included studies showed considerable variation. Bonaca et al. (2020) compared the outcomes in participants receiving rivaroxaban 2.5 mg BID plus aspirin 100 mg versus placebo plus aspirin [20]. Similarly, Zannad et al. (2018) compared rivaroxaban 2.5 mg BID plus aspirin or dual antiplatelet therapy to placebo plus aspirin or dual antiplatelet therapy [14]. In the Eikelboom et al. (2017) trial, rivaroxaban 2.5 mg BID and aspirin 100 mg daily was compared to either rivaroxaban alone or aspirin alone [13]. Dangas et al. (2020) compared a once-daily 10 mg dose of rivaroxaban combined with aspirin to dual antiplatelet therapy with aspirin and clopidogrel [15]. In the Mega et al. (2009) study, different doses and regimens of combined rivaroxaban and aspirin were compared to aspirin alone and aspirin with thienopyridine [11]. Mega et al. (2012) compared rivaroxaban 2.5 mg or 5 mg twice daily with low-dose aspirin to placebo plus aspirin [12]. The Yasuda et al. (2019) study tailored rivaroxaban dosing to renal function, providing either 10 or 15 mg once daily alongside aspirin or a P2Y12 inhibitor compared to rivaroxaban monotherapy [19]. Finally, Maximiliano et al. (2023) involved a higher dose (20 mg of rivaroxaban once daily alongside 300 mg aspirin) versus acenocoumarol [21]. Additionally, this study included patients with venous thromboembolism, which is out of the scope of this meta-analysis. Due to the differences in the study arms and the condition, this study was not included in this network meta-analysis. This study followed the VTE cases for 3 months, reporting three recurrent thromboembolic events in the acenocoumarol group compared to zero events in the rivaroxaban plus aspirin group. Additionally, minor bleeding occurred in five patients with acenocoumarol and zero with rivaroxaban plus aspirin. However, these differences lacked statistical significance, guiding for larger RCTs to further investigate this difference.

The remaining studies in this network meta-analysis primarily focused on a range of cardiovascular conditions. Two studies specifically included patients with symptomatic PAD [13,20]. One study focused exclusively on patients who had received transcatheter aortic valve replacement (TAVR) for treating aortic valve stenosis [15]. Coronary artery disease (CAD) served as an inclusion criterion in four studies, with one of these studies focusing exclusively on CAD patients who also had heart failure [14]. Additionally, one study enrolled patients with AF and stable CAD [19]. This study compared rivaroxaban alone to rivaroxaban plus one antiplatelet agent. The study concluded that rivaroxaban alone was as effective as combination therapy and offered better safety for patients with AF and stable CAD [19].

### 3.3. Quality Assessment

**Figure 2:**
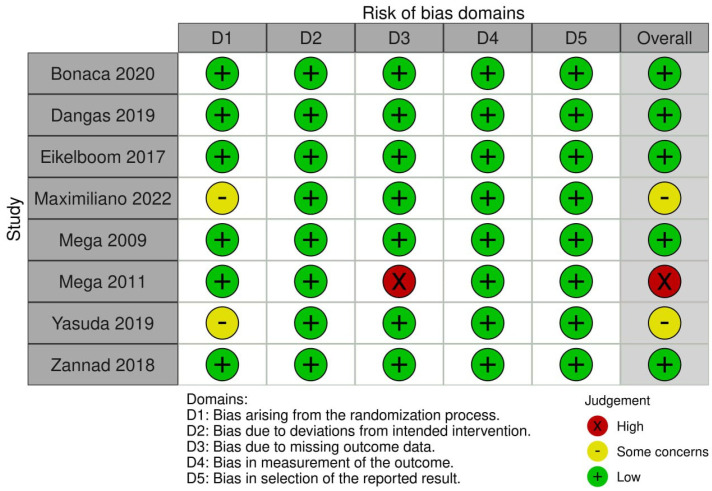
Risk of bias using (RoB-2).

The overall risk of bias was low in a total of five studies [11,13,14,15,20], moderate in two studies [19,21], and high in one study [12]. Details of risk of bias per each domain is demonstrated in Figure 2.

### 3.4. Meta-Analysis

#### 3.4.1. Risk of Thromboembolic Events

**Figure 3:**
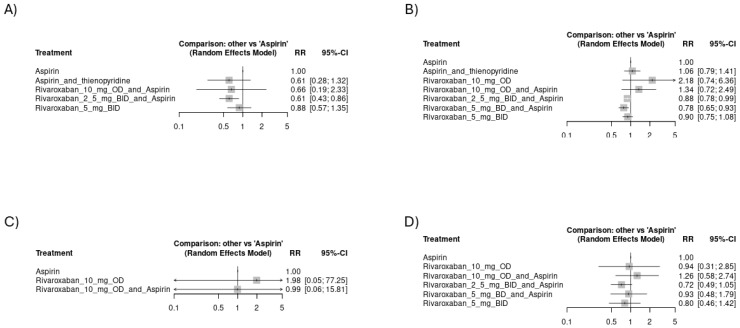
Forest plots showing the risk of thromboembolic events across study arms in all included studies (CVD): (**A**) risk of venous thromboembolic events, (**B**) risk of myocardial infarction events, (**C**) risk of systemic embolism, (**D**) risk of ischemic stroke.

**Figure 4:**
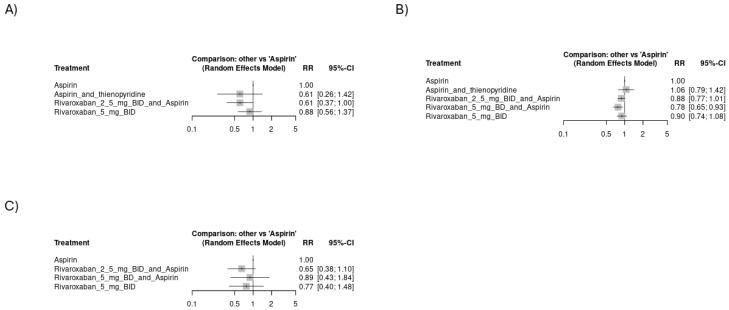
Forest plots showing the risk of thromboembolic events across study arms from studies focusing on CAD: (**A**) risk of venous thromboembolic events, (**B**) risk of myocardial infarction events, (**C**) risk of ischemic stroke.

Regarding venous thromboembolic events, the regimen of rivaroxaban 2.5 mg BID plus aspirin showed significant superiority over aspirin alone [RR = 0.61, CI 95% (0.43 to 0.86)]. Other arms showed better effectiveness in thromboembolic event prevention than aspirin alone, but lacking statistical significance (Figure 3A). The network plot is shown in Figure S1, while the league ranking table is provided in Table S1. Rivaroxaban 5 mg BID plus the aspirin group was associated with the least odds of myocardial infarction, followed by rivaroxaban 2.5 mg BID plus aspirin: [RR = 0.78, CI 95% (0.65 to 0.93)] and [RR = 0.88, CI 95% (0.78 to 0.99)], respectively (Figure 3B). The network plot is shown in Figure S2, while the league ranking table is provided in Table S2. We found no significant differences in the odds of systemic embolic events or ischemic stroke among the study arms. The network plot of systemic embolism is shown in Figure S3, while the league ranking table is provided in Table S3. However, rivaroxaban 2.5 mg BID plus aspirin was associated with the fewest odds of ischemic stroke (RR = 0.72), as indicated in Figure 3C,D. The network plot of ischemic stroke is shown in Figure S4, while the league ranking table is provided in Table S4. In the subset of studies including CAD, rivaroxaban 2.5 mg BID plus aspirin was associated with the least risk of venous thromboembolic events [RR = 0.61, CI 95% (0.37 to 1)] (Figure 4A). The network plot is shown in Figure S5, while the league ranking table is provided in Table S5. Rivaroxaban 5 mg BID plus the aspirin group was associated with the least risk of myocardial infarction [RR = 0.78, CI 95% (0.65 to 0.93)] (Figure 4B). The network plot is shown in Figure S6, while the league ranking table is provided in Table S6. No significant differences were observed regarding the risk of ischemic stroke among the study arms. However, rivaroxaban 2.5 mg BID plus aspirin had the lowest risk ratio (RR = 0.65), as indicated in (Figure 4C). The network plot is shown in Figure S7, while the league ranking table is provided in Table S7.

#### 3.4.2. Risk of Hemorrhagic Events

**Figure 5:**
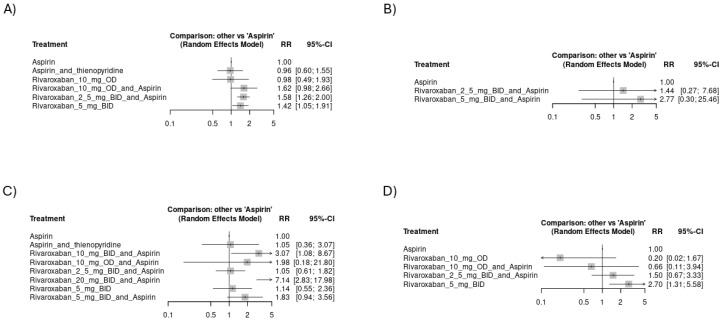
Forest plots showing the risk of hemorrhagic events across study arms in all included studies (CVD): (**A**) risk of major ISTH bleeding, (**B**) risk of TIMI major bleeding, (**C**) risk of fatal bleeding, (**D**) risk of hemorrhagic stroke.

**Figure 6:**
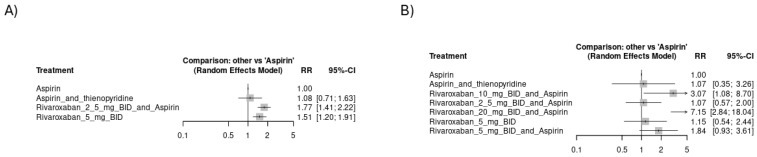
Forest plots showing the risk of hemorrhagic events across study arms from studies focusing on CAD: (**A**) risk of major ISTH bleeding, (**B**) risk of fatal bleeding.

Figure 5A shows that rivaroxaban 2.5 mg BID plus aspirin had the highest risk of major ISTH bleeding [RR = 1.58, CI 95% (1.26 to 2)], followed by rivaroxaban 10 mg OD with aspirin [RR = 1.62, CI 95% (0.98 to 2.66)] and Rivaroxaban 5 mg BID [RR = 1.42, CI 95% (1.05 to 1.91)]. The network plot is shown in Figure S8, while the league ranking table is provided in Table S8. Figure 5B shows no significant difference among study arms regarding TIMI major bleeding. The network plot is shown in Figure S9, while the league ranking table is provided in Table S9. Rivaroxaban 20 mg BID plus aspirin had the highest risk of fatal bleeding [RR = 7.14, CI 95% (2.83 to 17.98)], followed by rivaroxaban 10 mg BID plus aspirin [RR = 3.07, CI 95% (1.08 to 8.67)] and rivaroxaban 5 mg BID with aspirin [RR = 1.83, CI 95% (0.94 to 3.56)], as indicated in Figure 5C. The network plot is shown in Figure S10, while the league ranking table is provided in Table S10. Monotherapy with rivaroxaban 5 mg BID showed the highest risk of hemorrhagic stroke [RR = 2.7, CI 95% (1.31 to 5.58)], followed by rivaroxaban 2.5 mg BID with aspirin [RR = 1.5, CI 95% (0.67 to 3.33)], as shown in Figure 5D. The network plot is shown in Figure S11, while the league ranking table is provided in Table S11. Aspirin alone or aspirin with thienopyridine scored the safest among all study arms regarding bleeding events other than hemorrhagic stroke. A subgroup analysis of only the CAD studies yielded similar findings where rivaroxaban 2.5 mg BID with aspirin had the highest risk of major ISTH bleeding [RR = 1.77, CI 95% (1.41 to 2.22)], followed by rivaroxaban 5 mg BID [RR = 1.51, CI 95% (1.2 to 1.91)], as shown in Figure 6A. The network plot is shown in Figure S12, while the league ranking table is provided in Table S12. Similarly, rivaroxaban 20 mg BID with aspirin had the highest risk of fatal bleeding [RR = 7.15, CI 95% (2.84 to 18.04)], followed by rivaroxaban 10 mg BID with aspirin [RR = 3.07, CI 95% (1.08 to 8.7)] and rivaroxaban 5 mg BID with aspirin [RR = 1.84, CI 95% (0.93 to 3.61)], as indicated in Figure 6B. The network plot is shown in Figure S13, while the league ranking table is provided in Table S13.

#### 3.4.3. Mortality Events

**Figure 7:**
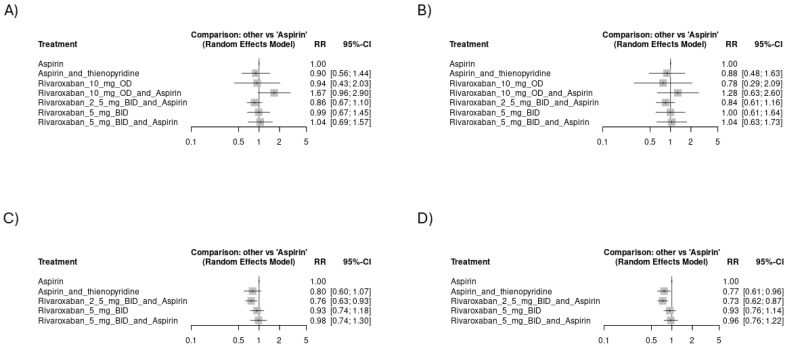
Forest plots showing the risk of mortality events: (**A**) risk of mortality events from any cause across study arms in all included studies (CVD), (**B**) risk of mortality events of CVS cause across study arms in all included studies (CVD), (**C**) risk of mortality events from any cause across study arms from the studies focusing on CAD, (**D**) risk of mortality events of CVS cause across study arms from the studies focusing on CAD.

Rivaroxaban 10 mg OD with aspirin had a higher tendency of mortality from any cause and mortality from a CVS cause: [RR = 1.67, CI 95% (0.96 to 2.9)] (Figure 7A) and [RR = 1.28, CI 95% (0.63 to 2.6)] (Figure 7B), respectively. However, these differences were just numerical with no statistical significance. The network plot of death from any cause is shown in Figure S14, while the league ranking table is provided in Table S14. The network plot of deaths from a CVS cause is shown in Figure S15, while the league ranking table is provided in Table S15. In a subgroup analysis of only the CAD studies, rivaroxaban 2.5 mg BID with aspirin had the lowest risk of mortality from any cause [RR = 0.76, CI 95% (0.63 to 0.93)] (Figure 7C). The network plot is shown in Figure S16, while the league ranking table is provided in Table S16. Regarding mortality of CVS cause, rivaroxaban 2.5 mg BID with aspirin had the lowest risk of mortality, followed by aspirin with thienopyridine: [RR = 0.73, CI 95% (0.62 to 0.87)] and [RR = 0.77, CI 95% (0.61 to 0.96)], as shown in Figure 7D. The network plot is shown in Figure S17, while the league ranking table is provided in Table S17.

## 4. Discussion

This network meta-analysis of seven RCTs pooled the current evidence regarding the safety and efficacy of different studied rivaroxaban regimens with or without aspirin in different CVD categories. We identified eight arms across the included studies: (aspirin + rivaroxaban 2.5 mg BID), (aspirin + rivaroxaban 10 mg OD), (aspirin and rivaroxaban 5 mg BID), (rivaroxaban 5 mg BID), (aspirin alone), (aspirin + thienopyridine), (rivaroxaban 10 mg OD), and (aspirin and rivaroxaban 20 mg BID). Aspirin alone was used as the reference comparator in all analyses. The rivaroxaban regimen of 2.5 mg BID combined with aspirin led to a 39% reduction in VTE risk compared to aspirin alone, with a risk ratio (RR) of 0.61. Additionally, both the 5 mg and 2.5 mg twice-daily doses of rivaroxaban, when combined with aspirin, lowered the risk of MI by 22% and 12%, respectively. All dosing strategies demonstrated similar effectiveness for ischemic stroke prevention, though the 2.5 mg BID plus aspirin showed a 22% reduction in stroke risk, which was not statistically significant. However, the 2.5 mg twice-daily rivaroxaban plus aspirin regimen significantly raised the risk of major bleeding by 58%. Monotherapy with 5 mg rivaroxaban BID also increased the risk of major bleeding and hemorrhagic stroke with risk ratios equal to 1.42 and 2.7, respectively. Two regimens were associated with significant increase in the risk of fatal bleeding: rivaroxaban 20 mg BID plus aspirin (RR = 7.14) and rivaroxaban 10 mg BID plus aspirin (3.7). Mortality events were comparable among all study arms with tendency towards a higher risk with rivaroxaban 10 mg once daily plus aspirin. A separate analysis of four studies focusing on coronary artery diseases (CAD) was conducted. Similarly, the regimen of rivaroxaban 5 mg BID with aspirin decreased the risk of MI by 22%. The regimen of rivaroxaban 2.5 mg BID plus aspirin decreased the risk of VTE by 39%. The risk of major bleeding remained significantly higher with rivaroxaban 2.5 mg BID plus aspirin and monotherapy of rivaroxaban 5 mg BID. Similarly, the risk of fatal bleeding remained the highest with rivaroxaban 20 mg BID plus aspirin and rivaroxaban 10 mg BID plus aspirin. Regarding the mortality risk in the CAD studies, rivaroxaban 2.5 mg BID plus aspirin reduced the risk of death by 24% when compared to aspirin monotherapy.

Incorporating anticoagulants into prophylaxis regimens following acute coronary syndrome (ACS) is often justified by providing additional protection against further thromboembolic events. However, this practice is always limited by the increased risk of bleeding, which must be carefully balanced against the benefits of preventing further cardiovascular events. A previous meta-analysis of 14 RCTs compared warfarin plus aspirin to aspirin alone after the (ACS). They reported that aspirin plus warfarin, targeting an international normalized ratio (INR) to 2–3, decreased the risk of stroke and MI by 34% and 40%, respectively. However, the risk of major bleeding increased significantly by 74% [22]. Another recent network meta-analysis of six RCTs compared non-vitamin K antagonist oral anticoagulants (NOACs) plus aspirin to aspirin alone. The analysis suggested that rivaroxaban decreases the incidence of all-cause mortality and probably also the incidence of deaths attributed to cardiovascular causes. Rivaroxaban and apixaban, but not dabigatran, increased the risk of major bleeding compared with a placebo [23]. Mega et al. [11] conducted the first trial investigating the use of rivaroxaban after acute coronary syndrome. They compared the safety and efficacy outcomes of rivaroxaban given at different total daily doses from 5 to 20 mg versus aspirin alone. The authors reported that, while rivaroxaban may reduce major ischemic events, this benefit is offset by an increase of risk of bleeding in a dose-dependent manner. Another RCT by Mega et al. investigated the twice doses of either 2.5 mg or 5 mg of rivaroxaban versus placebo. They observed that rivaroxaban was associated with less odds of MI, stroke, and death from cardiovascular causes. However, it was associated with the increased risk of major bleeding and intracranial hemorrhage with the comparable risk of fatal bleeding [12]. Similarly, Eikelboom et al. reported that, in patients with stable atherosclerotic vascular disease, rivaroxaban 2.5 mg twice daily plus aspirin was associated with better efficacy outcomes and worse safety in terms of the increased event of major bleeding than those assigned to aspirin alone. Additionally, they observed that monotherapy with rivaroxaban 5 mg twice daily had comparable outcomes with aspirin but with more major bleeding events [13]. Zannad et al. investigated rivaroxaban in patients with worsening heart failure with CAD. This study found no significant reduction in the risk of stroke, MI, rehospitalization for heart failure, or death when rivaroxaban at a dose of 2.5 mg twice daily plus aspirin was compared to aspirin alone in this group of patients [14].

Bonaca et al. included patients with PAD after revascularization, reporting that rivaroxaban 2.5 mg BID plus aspirin had lower odds of MI, stroke, acute limb ischemia, and death than aspirin alone. There was a significant difference in major bleeding events as defined by the ISTH bleeding scale, but no difference was observed using the TIMI major bleeding scale. The ISTH scale has broader applicability across various bleeding-related disorders, which likely accounts for its higher sensitivity in detecting bleeding risks, highlighting a difference that the TIMI scale did not capture [20,24]. Dangas et al. compared the combination of daily 10 mg of rivaroxaban plus aspirin to dual antiplatelet therapy in cases which underwent TAVR without established indication for anticoagulation. This study reported both worse safety and efficacy outcomes with the combination regimen. These findings point to the importance of balancing between efficacy and safety when selecting antithrombotic therapy regimens. Research is ongoing to optimize the dose of rivaroxaban in each category separately [25,26,27]. Finally, the decision should be based on individual bleeding and thromboembolic risks rather than a one-size-fits-all approach. Rivaroxaban 5 mg BID and 2.5 mg BID plus aspirin increased the risk of major ISTH bleeding by 42% and 52%, respectively, while combining rivaroxaban 10 mg OD with aspirin raised this risk by 62%. TIMI major bleeding rose significantly—by 144% and 177%—with rivaroxaban 2.5 mg BID plus aspirin and 5 mg BID plus aspirin, respectively. Fatal bleeding was most pronounced with rivaroxaban 20 mg BID plus aspirin (714% increase), followed by 198% and 105% increases with rivaroxaban 10 mg OD alone and in combination with aspirin, respectively. Hemorrhagic stroke risk increased by 270% with rivaroxaban 5 mg BID and 50% with the 2.5 mg BID plus aspirin regimen. These data underscore that the true clinical value of any regimen must be based on a careful evaluation of both its cardiovascular benefits and bleeding risks to assess net clinical benefit. Supporting this, real-world studies revealed that rivaroxaban 10 mg once daily was associated with significantly lower bleeding risk (3.7% vs. 14.7%) compared to higher doses (15–20 mg daily), suggesting that lower-dose regimens may offer a more favorable safety–efficacy balance in high-risk populations [17,26].

This network meta-analysis comprehensively pooled the available data regarding different regimens of rivaroxaban with and without aspirin through direct and indirect comparisons to inform the future research, focusing on the safest regimens. This study has some limitations. The included studies were heterogeneous in terms of patient populations, which was addressed by conducting a subgroup analysis on the CAD studies. However, we could not perform similar analyses in other categories due to the unavailability of enough studies. Additionally, many of the included studies followed the cases for a short time, limiting the ability to consistently assess the long-term outcomes. Moreover, as a network meta-analysis, it relies on both direct and indirect comparisons, which can sometimes yield contradictory evidence and challenge the consistency of the findings. Smaller studies included in the analysis may also exaggerate or overestimate the treatment effects, potentially skewing the results. Another limitation lies in the variability of the definitions and endpoints across the included studies, such as the criteria for major bleeding or thrombotic events, which could introduce inconsistency in outcome measures. The quality of the included studies, though they were assessed, varied, with some studies being rated as having a moderate or high risk of bias, which could influence the robustness of the findings. Furthermore, differences in baseline characteristics and comorbidities across studies may influence both the efficacy and safety outcomes, thereby affecting the generalizability of the results. We believe that the findings of this network meta-analysis provide valuable general insights into the comparative effectiveness of antithrombotic regimens. However, due to heterogeneity in patient populations and comorbid conditions, caution is warranted in applying these results universally. Therefore, additional network meta-analyses focusing on specific disease categories and stratified populations are necessary to guide more tailored clinical decision-making. There remains a notable gap in the literature regarding head-to-head comparisons of different rivaroxaban regimens. We acknowledge that this gap limits our understanding of the optimal rivaroxaban regimen for various patient populations, and further head-to-head trials would be beneficial to directly compare the efficacy and safety profiles of different rivaroxaban dosages and regimens. Additionally, another gap exists in the representation of certain populations, particularly those with PAD, AF with CAD, VTE, and prosthetic valve patients. Further studies are needed to explore rivaroxaban regimens in these underrepresented populations. Finally, comparative studies with different anticoagulants are also warranted. We believe these gaps should be addressed in future research to refine treatment protocols and improve the generalizability of findings across broader patient cohorts.

## 5. Conclusions

In patients with CVD at risk of thromboembolic complications, the regimen of rivaroxaban 2.5 mg BID plus aspirin is effective in preventing VTE while also presenting the highest risk of major bleeding. In patients with CAD, this regimen showed the lowest mortality risk. Rivaroxaban 5 mg BID plus aspirin showed the lowest risk of MI, followed by rivaroxaban 2.5 mg BID plus aspirin. This network meta-analysis highlighted that, while combined therapy might be effective in certain populations, its safety profile—specifically the increased risk of bleeding—raises questions about its widespread use.

## Figures and Tables

**Table 1:** Summary of the included studies.

Study ID	Study Time and Sites	Protocol Number	Total Number of Patients	Disease Details	Rivaroxaban Arm Details	Control Arm Details	Additional Therapy Details	Follow up Duration (months)	Primary Outcome
Bonaca et al. (2020) [20]	2015–2018 34 countries	NCT02504216	6564	Type of disease: moderate to severe symptomatic lower extremity atherosclerotic peripheral artery disease. Participant requirements: they should have undergone successful peripheral revascularization distal to the external iliac artery for symptomatic PAD (peripheral artery disease) within 10 days prior to randomization.	Twice daily oral Rivaroxaban 2.5 mg and aspirin 100 mg daily	Placebo plus aspirin	Clopidogrel may be administered for up to 6 months following revascularization at the discretion of the investigator.	28	The primary measure of effectiveness was a combination of acute limb ischemia, major amputation due to vascular issues, myocardial infarction, ischemic stroke, or death from cardiovascular causes. The main focus on safety was the occurrence of major bleeding, as defined by the thrombolysis in myocardial infarction (TIMI) classification.
Dangas et al. (2020) [15]	2015–2018 16 countries	NCT02556203	1644	Type of disease: transcatheter aortic valve replacement (TAVR) for the treatment of aortic valve stenosis. Patient requirements: the patient should have undergone a successful TAVR, which is defined as the proper placement of an approved bioprosthetic aortic valve into the correct anatomical location, with the valve functioning as intended and without any complications during the procedure.	Once daily oral 10 mg rivaroxaban and aspirin (75–100 mg).	Oral 75–100 mg of aspirin and 75 mg of clopidogrel daily.	NR	3	The primary measure of effectiveness was a combination of death from any cause or thromboembolic events, such as stroke, heart attack, symptomatic valve thrombosis, systemic embolism (not involving the central nervous system), deep-vein thrombosis, or pulmonary embolism. The main focus on safety was a combination of life-threatening, disabling, or major bleeding.
Eikelboom et al. (2017) [13]	2013–2016 33 countries	NCT01776424	27395	Type of disease: coronary artery disease (CAD) and/or peripheral artery disease (PAD). CAD: one of the following: - myocardial infarction within the past 20 years - multi-vessel coronary artery disease - history of stable or unstable angina - previous multi-vessel percutaneous coronary intervention - previous multi-vessel coronary artery bypass graft surgery. PAD: one of the following: - previous aorto-femoral bypass surgery, limb bypass surgery, or percutaneous transluminal angioplasty revascularization of the iliac, or infra-inguinal arteries - previous limb or foot amputation for arterial vascular disease - history of intermittent claudication and one or more of the following: (1) ankle/arm blood pressure (BP) ratio < 0.90 (2) significant peripheral artery stenosis (≥50%) documented by angiography or duplex ultrasound - previous carotid revascularization or asymptomatic carotid artery stenosis ≥50% as diagnosed by duplex ultrasound or angiography	Twice daily oral Rivaroxaban 2.5 mg and aspirin 100 mg daily.	Group 1 rivaroxaban alone (5 mg orally twice a day). Group 2 aspirin alone (100 mg orally once a day).	NR	23	The primary measure of effectiveness was a combination of the first instance of stroke, heart attack, or cardiovascular death. The main focus on safety was significant bleeding, which was defined as fatal bleeding, symptomatic bleeding into a critical organ or area, bleeding at the site of surgery requiring further operation, or bleeding requiring a visit or admission to the hospital.
Maximiliano et al. (2023) [21]	2021–2022 Mexico	NCT05515120	62	Type of disease: venous thromboembolism. Participant requirements: individuals should meet the following criteria: (I) have experienced a confirmed initial episode of venous thromboembolism, such as upper extremity deep vein thrombosis, proximal lower extremity deep vein thrombosis, or pulmonary embolism; (II) have documented recurrent venous thromboembolism, including new or extended upper extremity deep vein thrombosis, proximal lower extremity deep vein thrombosis, or pulmonary embolism, while receiving systemic anticoagulation treatment (specifically rivaroxaban 20 mg once a day).	Once daily 20 mg of oral rivaroxaban and 300 mg of aspirin	Acenocoumarol oral tablet.	NR	3	The primary measure of effectiveness was the presence of thromboembolic events (recurrent ipsilateral DVT, recurrent contralateral DVT, PE, ischemic stroke, and myocardial infarction). The main focus on safety was minor bleeding according to the International Society on Thrombosis and Hemostasis (ISTH) scale.
Mega et al. (2009) [11]	2006–2008 27 countries	NCT00402597	3491	Type of disease: acute coronary syndrome. Patient requirements: patients should have experienced symptoms suggestive of an acute coronary syndrome lasting at least 10 min at rest. This includes a diagnosis of ST-elevation myocardial infarction (STEMI) or a diagnosis of non-STEMI or unstable angina with specific indicators, such as raised cardiac enzyme markers, 1 mm or more ST-segment deviation, or a TIMI risk score of 3 or more	Rivaroxaban 5 mg orally plus aspirin (75–100) mg. Rivaroxaban 10 mg orally plus aspirin (75–100) mg. Rivaroxaban 20 mg orally plus aspirin (75–100) mg. Group 1: once daily. Group 2: twice daily (the same total daily dose).	Group 1: oral aspirin (75–100) mg. Group 2: oral rivaroxaban (5, 10, 15, 20) mg, aspirin (75–100) mg, and thienopyridine. Group 3: oral aspirin (75–100) mg and thienopyridine	According to the use of thienopyridine, the group was divided into two strata (stratum one received aspirin only, while stratum two received aspirin plus a thienopyridine).	6	The primary measure of effectiveness was the time to the first occurrence of death, myocardial infarction, stroke, or severe recurrent ischemia requiring revascularization within 6 months from enrollment. The main focus on safety was clinically significant bleeding, defined as TIMI major, TIMI minor, or requiring medical attention. Bleeding requiring medical attention was further defined as a bleeding event that necessitated medical treatment, surgical treatment, or laboratory assessment, and did not meet criteria for TIMI major or minor bleeding.
Mega et al. (2012) [12]	2008–2011 44 countries	NCT00809965	15526	Type of Disease: acute coronary syndrome. Participant requirements: - must have experienced either ST-segment elevation myocardial infarction (STEMI), non–ST-segment elevation myocardial infarction (NSTEMI), or unstable angina - patients under 55 years of age who have either diabetes mellitus or a prior myocardial infarction along with the index event.	Group 1: twice daily of oral rivaroxaban 2.5 mg and low dose aspirin. Group 2: twice daily of oral rivaroxaban 5 and low dose aspirin.	Placebo plus low dose aspirin.	Patients were to receive thienopyridine (either clopidogrel or ticlopidine) according to the national or local guidelines.	31	The primary measure of effectiveness was a combination of death from cardiovascular causes, heart attack, or stroke (either caused by blockage of blood vessels or bleeding in the brain). The main focus on safety was major bleeding not related to coronary artery bypass surgery (CABG).
Yasuda et al. (2019) [19]	2015–2017 Japan	NCT02642419	2240	Type of disease: atrial fibrillation and stable coronary artery disease. Participant requirements: they should have a score of at least 1 on the CHADS2 scale. Additionally, patients should meet at least one of the following criteria: - a history of PCI, including angioplasty with or without stenting, at least 1 year before enrollment - a history of angiographically confirmed coronary artery disease (with stenosis of ≥50%) not requiring revascularization - a history of coronary-artery bypass grafting (CABG) at least 1 year before enrollment.	Once daily oral 10 mg rivaroxaban if creatinine clearance (15–49) per minute plus aspirin, or 15 mg if creatinine clearance of ≥50 mL per minute	Once daily oral 10 mg rivaroxaban if creatinine clearance (15–49) per minute, or 15 mg if creatinine clearance of ≥50 mL per minute.	Patients in the combination group may take either aspirin or a P2Y12 inhibitor, according to the discretion of the treating physician.	24.1	The primary measure of effectiveness was a combination of stroke, systemic embolism, myocardial infarction, unstable angina requiring revascularization, or death from any cause. The main focus on safety was the occurrence of major bleeding, as per the criteria established by the International Society on Thrombosis and Hemostasis.
Zannad et al. (2018) [14]	2013–2017 32 countries	NCT01877915	5022	Type of disease: heart failure. Participant requirements: participants should have a history of chronic heart failure lasting at least 3 months, a left ventricular ejection fraction of 40% or less, and coronary artery disease, and those have been treated for an episode of worsening heart failure within the past 21 days. Additionally, their plasma concentration of brain natriuretic peptide (BNP) should be at least 200 pg. per milliliter, or their N-terminal pro-brain natriuretic peptide (NT-proBNP) should be at least 800 pg. per milliliter	Twice daily oral rivaroxaban 2.5 mg and aspirin.	Placebo plus (aspirin or dual antiplatelet).	Single or dual antiplatelet therapy was allowed.	21.1	The primary measure of effectiveness was a combination of death from any cause, myocardial infarction, or stroke. Additional measures of effectiveness included death from cardiovascular causes, rehospitalization for worsening heart failure, and the combination of death from any cause or rehospitalization for worsening heart failure. The main focus on safety was a combination of fatal bleeding or bleeding into a critical space with the potential for causing permanent disability.

**Table 2:** Baseline characteristics of the included studies.

Study ID	Study Groups (n)	Age, Mean (SD)	Sex (Male), No. (%)	BMI, Mean (SD)	E-GFR, Mean (SD)	Hypertension, No. (%)	Current Smoking, No. (%)	Diabetes, No. (%)	Prior MI, No. (%)	Stroke, No. (%)	Dyslipidemia, No. (%)
Bonaca et al. (2020) [20]	Rivaroxaban 2.5 mg group (3286)	67 (8.8)	2439 (74.2%)	26.1 (4.3)	NR	2684 (81.7%)	1147 (39.3%)	1313 (40%)	365 (11.1%)	NR	1971 (60%)
	Aspirin group (3278)	67 (8.8)	2421 (73.8%)	26.1 (4.3)	NR	2658 (81.1%)	1132 (34.5%)	1316 (40.1%)	349 (10.6%)	NR	1968 (60%)
Dangas et al. (2020) [15]	Rivaroxaban 10 mg group (826)	80.4 (7.1)	426 (51.6%)	28.1 (5.5)	73.4 (23.8)	720 (82.2%)	NR	236 (28.6%)	NR	51 (6.20%)	NR
	Aspirin group (818)	80.8 (6)	405 (49.5%)	28.2 (5.7)	73.2 (23.3)	697 (85.2%)	NR	235 (28.7%)	NR	35 (4.3%)	NR
Eikelboom et al. (2017) [13]	Rivaroxaban 2.5 mg group (9152)	68.3 (7.9)	7093 (77.5%)	28.3 (4.8)	NR	6907 (75.5%)	1944 (21.2%)	3448 (37.7%)	5654 (61.8%)	351 (3.8%)	NR
	Rivaroxaban 5 mg only group (9117)	68.2 (7.9)	7145 (88.4%)	28.3 (4.6)	NR	6848 (75.1%)	1451 (21.4%)	3419 (37.5%)	5653 (62%)	346 (3.8%)	NR
	Aspirin group (9126)	68.2 (8)	7137 (88.2%)	28.4 (4.7)	NR	6677 (75.4%)	1972 (21.6%)	3474 (38.1%)	5721 (62.2%)	335 (3.7%)	NR
Maximiliano et al. (2023) [21]	Rivaroxaban 20 mg group (28)	42.89 (15.33)	11 (39.29%)	26.2	97.5 (3.2)	8 (28.57%)	2 (7.14%)	8 (28.57%)	NR	NR	NR
	Acenocoumarol group (30)	42.76 (15.58)	10 (33.3%)	25.8	92.9 (14.3)	5 (16.66%)	4 (13.3%)	9 (30%)	NR	NR	NR
Mega et al. (2009) [11]	Rivaroxaban (5, 10, 20 mg) groups (508)	59.8 (9.2)	340 (66.9%)	28.5 (4.8)	70.1 (31.1)	387 (76.2%)	243 (47.8%)	102 (20.1%)	141 (27.8%)	NR	241 (47.4%)
	Aspirin group (253)	60.3 (9.3)	182 (68%)	28.1 (4.7)	79.3 (27.7)	188 (74.3%)	129 (51%)	55 (21.7%)	70 (27.7%)	NR	121 (44.3%)
	Rivaroxaban, aspirin and thienopyridine group (1823)	56.5 (9.5)	1470 (80.6%)	28.8 (5.9)	102.4 (31.5)	951 (52.2%)	1199 (65.8%)	350 (19.2%)	345 (18.9%)	NR	795 (43.6%)
	Aspirin and thienopyridine group (907)	57.1 (9.5)	713 (78.6%)	28.4 (4.7)	99.9 (31.7)	471 (52%)	596 (65.8%)	166 (18.3%)	180 (19.9%)	NR	393 (43.4%)
Mega et al. (2012) [12]	Rivaroxaban 2.5 mg group (5174)	61.8 (9.2)	3875 (74.9%)	NR	86.13 (27.7)	3470 (67.1%)	NR	1669 (31.31%)	1363 (26.3%)	NR	2448 (48.3%)
	Rivaroxaban 5 mg group (5176)	61.9 (9)	3843 (43.2%)	NR	86 (26.8)	3499 (67.6%)	NR	1648 (31.8%)	1403 (27.1%)	NR	2544 (49.1%)
	Aspirin group (5176)	61.5 (9.4)	3882 (85%)	NR	86.4 (27.1)	3494 (67.5%)	NR	1647 (31.8%)	1415 (27.3%)	NR	2496 (48.7%)
Yasuda et al. (2019) [19]	Rivaroxaban 10 mg group (1107)	74.4 (8.2)	876 (79.1%)	24.5 (3.7)	61.7 (24)	NR	146 (13.2%)	466 (42.1%)	393 (35.5%)	175 (15.8%)	NR
	Aspirin group (1108)	74.3 (8.3)	875 (79%)	24.5 (3.7)	62.8 (25.8)	NR	146 (13.7%)	461 (41.6%)	384 (34.7%)	148 (13.4%)	NR
Zannad et al. (2018) [14]	Rivaroxaban 2.5 mg group (2507)	66.5 (10.1)	1956 (78%)	27.6 (5.1)	NR	1897 (25.7%)	NR	1024 (40.8%)	1911 (76.2%)	208 (8.3%)	NR
	Aspirin group (2515)	66.3 (10.3)	1916 (76.2%)	27.8 (5.3)	NR	1886 (75%)	NR	1028 (40.9%)	1892 (75.2%)	245 (9.7%)	NR

## Data Availability

No new data were created or analyzed in this study.

## Supplementary Materials

The following supporting information can be downloaded at: https://www.mdpi.com/article/10.3390/jcm14103437/s1.
