# Supplementary material for: Outcomes of Different Regimens of Rivaroxaban and Aspirin in Cardiovascular Diseases: A Network Meta-Analysis
Source: J Clin Med. 2025 May 14;14(10):3437. doi: 10.3390/jcm14103437 (PMC12112727; doi:10.3390/jcm14103437)
Supplement: Supplementary file 1 [file jcm-14-03437-s001.zip › jcm-3616240-supplementary.pdf]

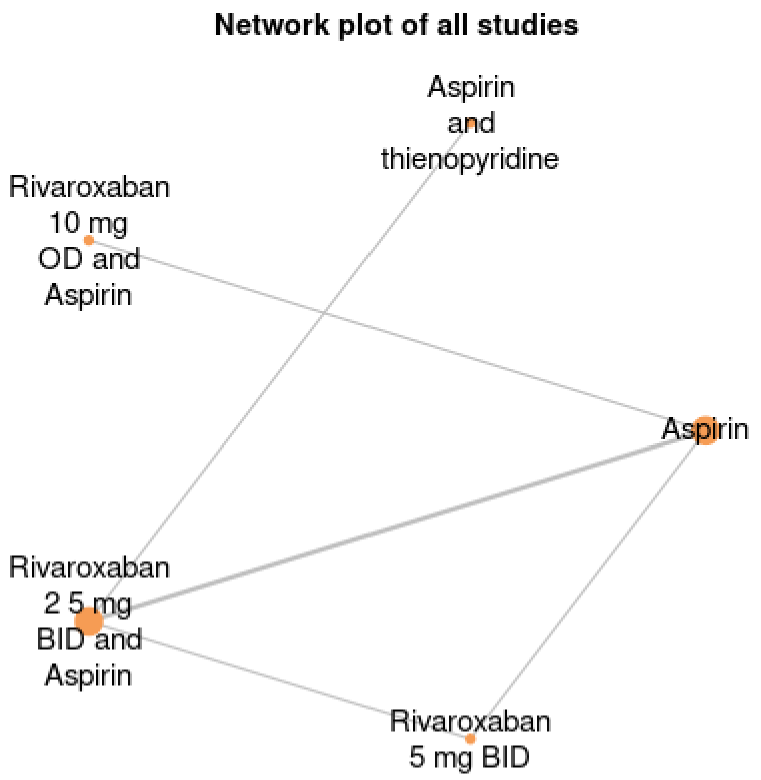

Fig.S1.: Venous thromboembolism (all included studies)

Rivaroxaban_2_5_mg_BID_and_Aspirin				
1.00 [0.50; 2.00]	Aspirin_and_thienopyridine			
0.92 [0.25; 3.41]	0.92 [0.21; 4.03]	Rivaroxaban_10_mg_OD_and_Aspirin		
0.69 [0.44; 1.10]	0.69 [0.30; 1.58]	0.75 [0.20; 2.84]	Rivaroxaban_5_mg_BID	
0.61 [0.43; 0.86]	0.61 [0.28; 1.32]	0.66 [0.19; 2.33]	0.88 [0.57; 1.35]	Aspirin

Table.S1.: Venous thromboembolism (all included studies)

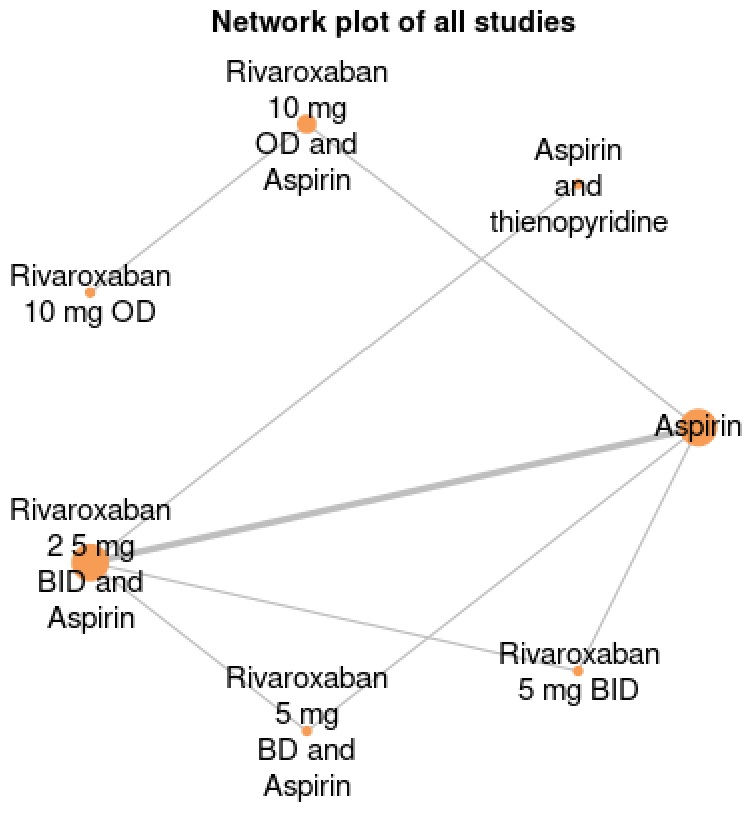

Fig.S2.: Myocardial infarction (all included studies)

Rivaroxaban_5_mg_BD_and_Aspirin						
0.88 [0.73; 1.06]	Rivaroxaban_2_5_mg_BID_and_Aspirin					
0.87 [0.68; 1.11]	0.98 [0.82; 1.18]	Rivaroxaban_5_mg_BID				
0.78 [0.65; 0.93]	0.88 [0.78; 0.99]	0.90 [0.75; 1.08]	Aspirin			
0.73 [0.53; 1.01]	0.83 [0.64; 1.08]	0.85 [0.61; 1.17]	0.94 [0.71; 1.26]	Aspirin_and_thienopyridine		
0.58 [0.30; 1.10]	0.66 [0.35; 1.24]	0.67 [0.35; 1.28]	0.75 [0.40; 1.39]	0.79 [0.40; 1.56]	Rivaroxaban_10_mg_OD_and_Aspirin	
0.36 [0.12; 1.06]	0.41 [0.14; 1.19]	0.41 [0.14; 1.22]	0.46 [0.16; 1.34]	0.49 [0.16; 1.48]	0.62 [0.26; 1.48]	Rivaroxaban_10_mg_OD

Table S2.: Myocardial infarction (all included studies)

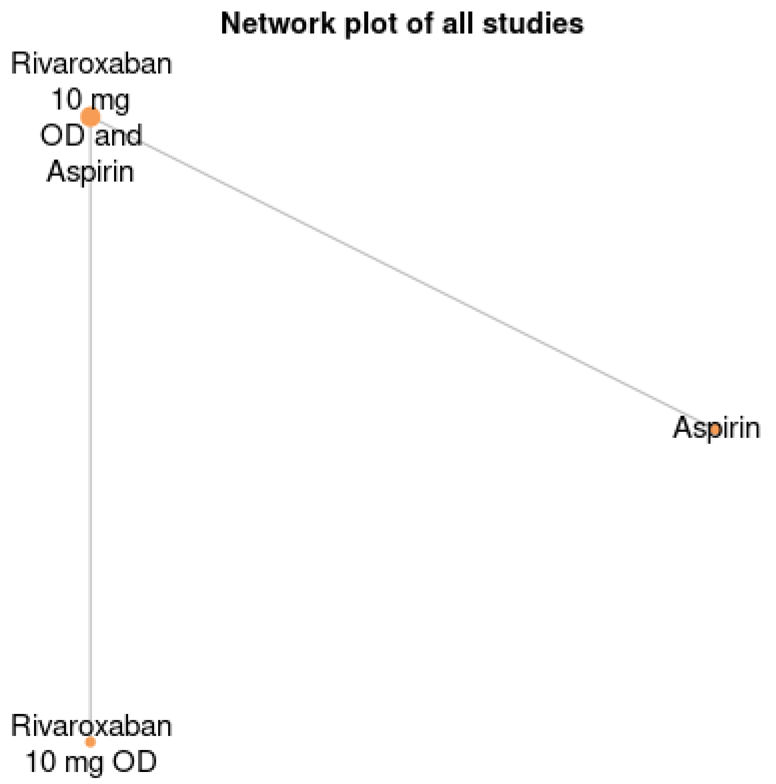

Fig.S3.: Systemic embolism (all included studies)

Rivaroxaban_10_mg_OD_and_Aspirin		
0.99 [0.06; 15.81]	Aspirin	
0.50 [0.05; 5.51]	0.51 [0.01; 19.73]	Rivaroxaban_10_mg_OD

Table S3.: Systemic embolism (all included studies)

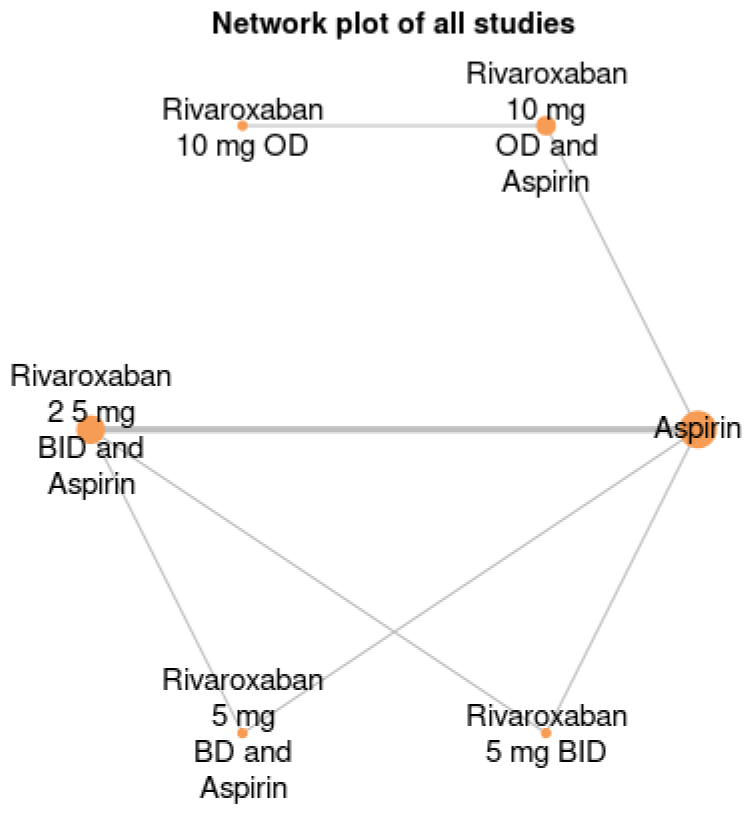

Fig.S4.: Ischemic stroke (all included studies)

Rivaroxaban_2_5_mg_BID_and_Aspirin					
0.89 [0.50; 1.58]	Rivaroxaban_5_mg_BID				
0.76 [0.24; 2.43]	0.85 [0.25; 2.95]	Rivaroxaban_10_mg_OD			
0.77 [0.40; 1.49]	0.87 [0.38; 1.98]	1.02 [0.28; 3.67]	Rivaroxaban_5_mg_BD_and_Aspirin		
0.72 [0.49; 1.05]	0.80 [0.46; 1.42]	0.94 [0.31; 2.85]	0.93 [0.48; 1.79]	Aspirin	
0.57 [0.24; 1.35]	0.64 [0.24; 1.67]	0.75 [0.34; 1.64]	0.74 [0.27; 2.04]	0.79 [0.36; 1.73]	Rivaroxaban_10_mg_OD_and_Aspirin

Table S4.: Ischemic stroke (all included studies)

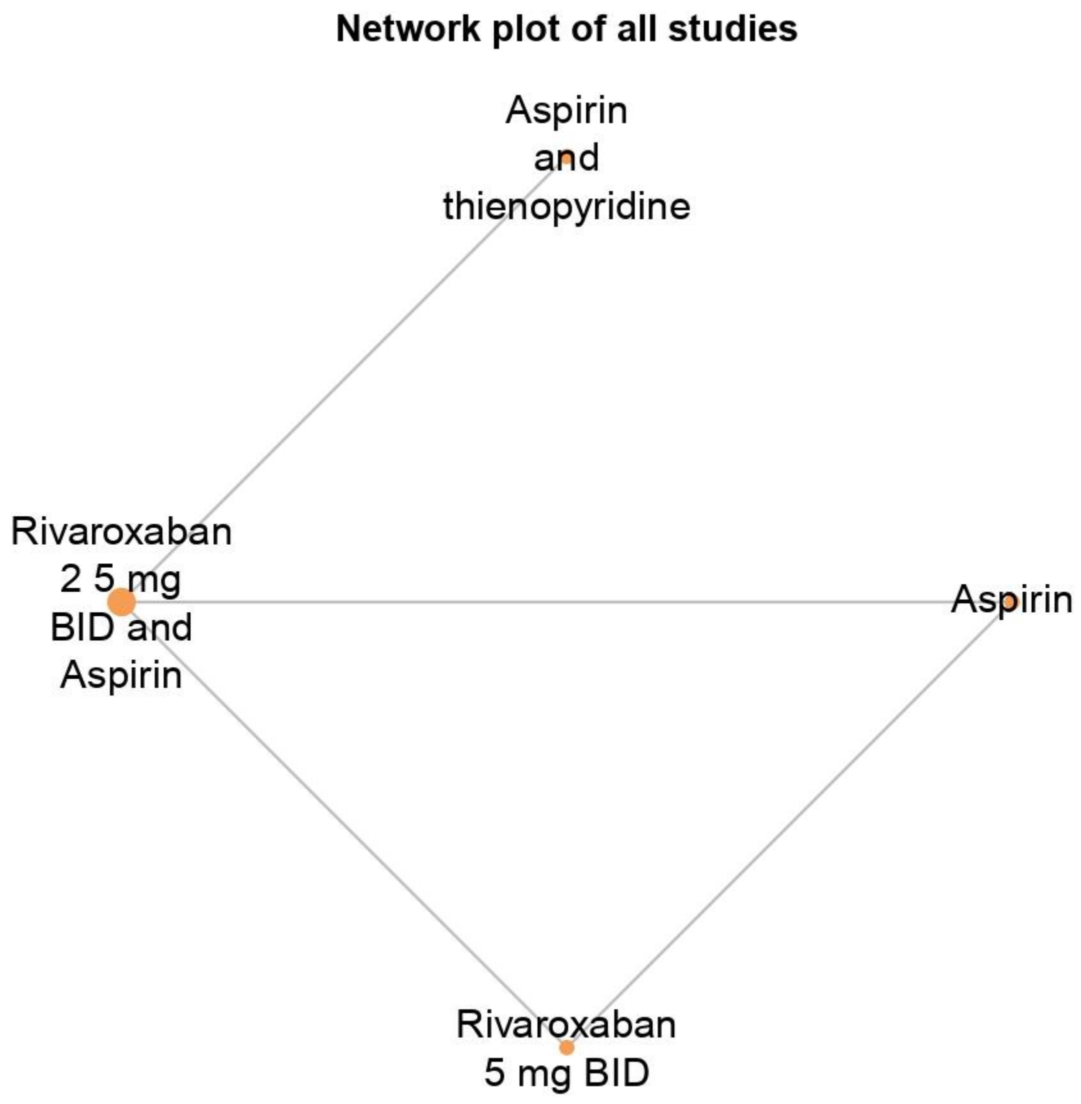

Fig.S5.: Venous thromboembolism (CAD studies)

Rivaroxaban_2_5_mg_BID_and_Aspirin			
1.00 [0.50; 2.00]	Aspirin_and_thienopyridine		
0.69 [0.42; 1.15]	0.69 [0.29; 1.63]	Rivaroxaban_5_mg_BID	
0.61 [0.37; 1.00]	0.61 [0.26; 1.42]	0.88 [0.56; 1.37]	Aspirin

Table S5.: Venous thromboembolism (CAD studies)

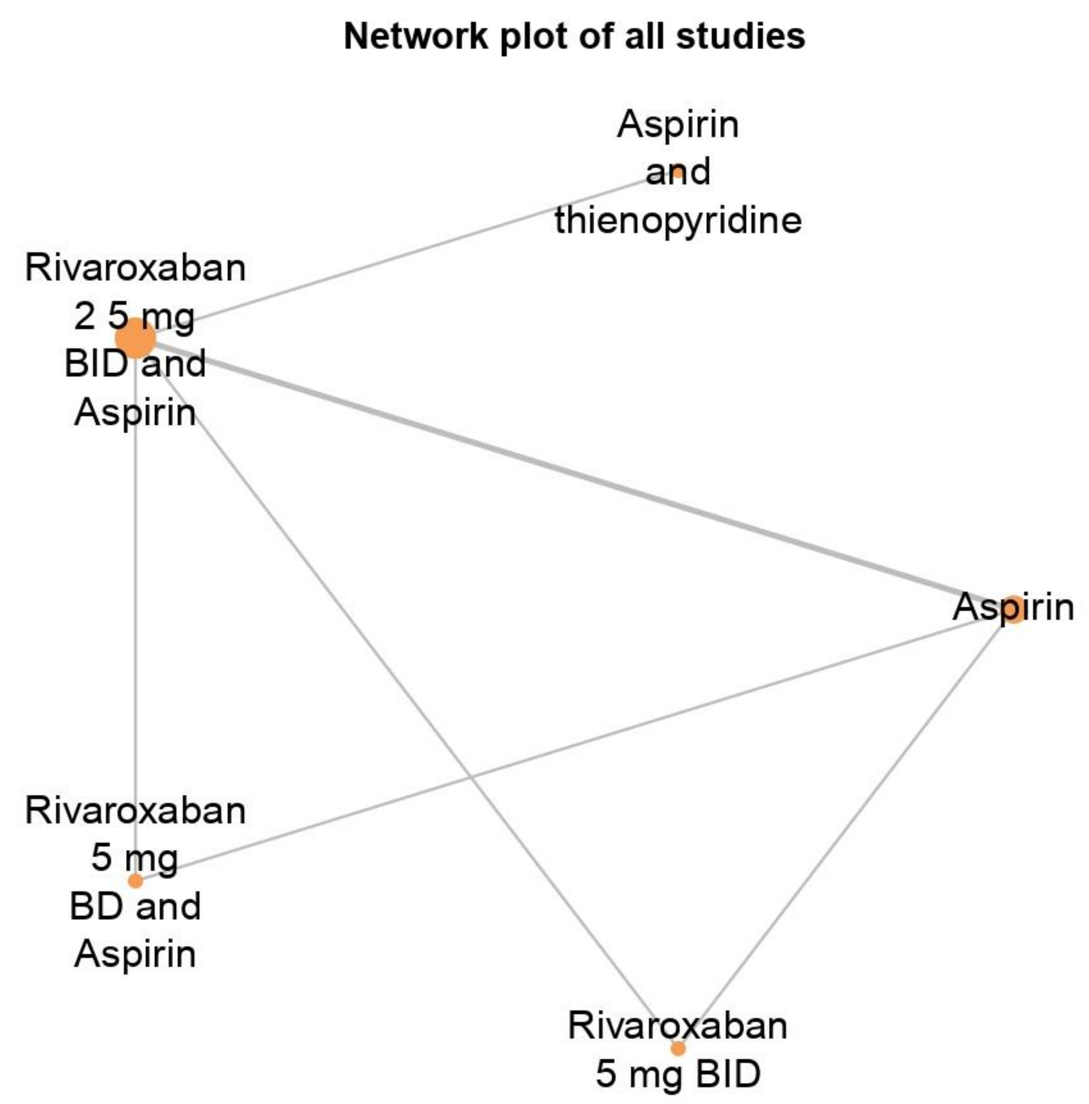

Fig.S6.: Myocardial infarction (CAD studies)

Rivaroxaban_5_mg_BD_and_Aspirin				
0.88 [0.73; 1.06]	Rivaroxaban_2_5_mg_BID_and_Aspirin			
0.87 [0.68; 1.11]	0.98 [0.81; 1.19]	Rivaroxaban_5_mg_BID		
0.78 [0.65; 0.93]	0.88 [0.77; 1.01]	0.90 [0.74; 1.08]	Aspirin	
0.73 [0.53; 1.01]	0.83 [0.64; 1.08]	0.85 [0.61; 1.17]	0.95 [0.70; 1.27]	Aspirin_and_thienopyridine

Table S6.: Myocardial infarction (CAD studies)

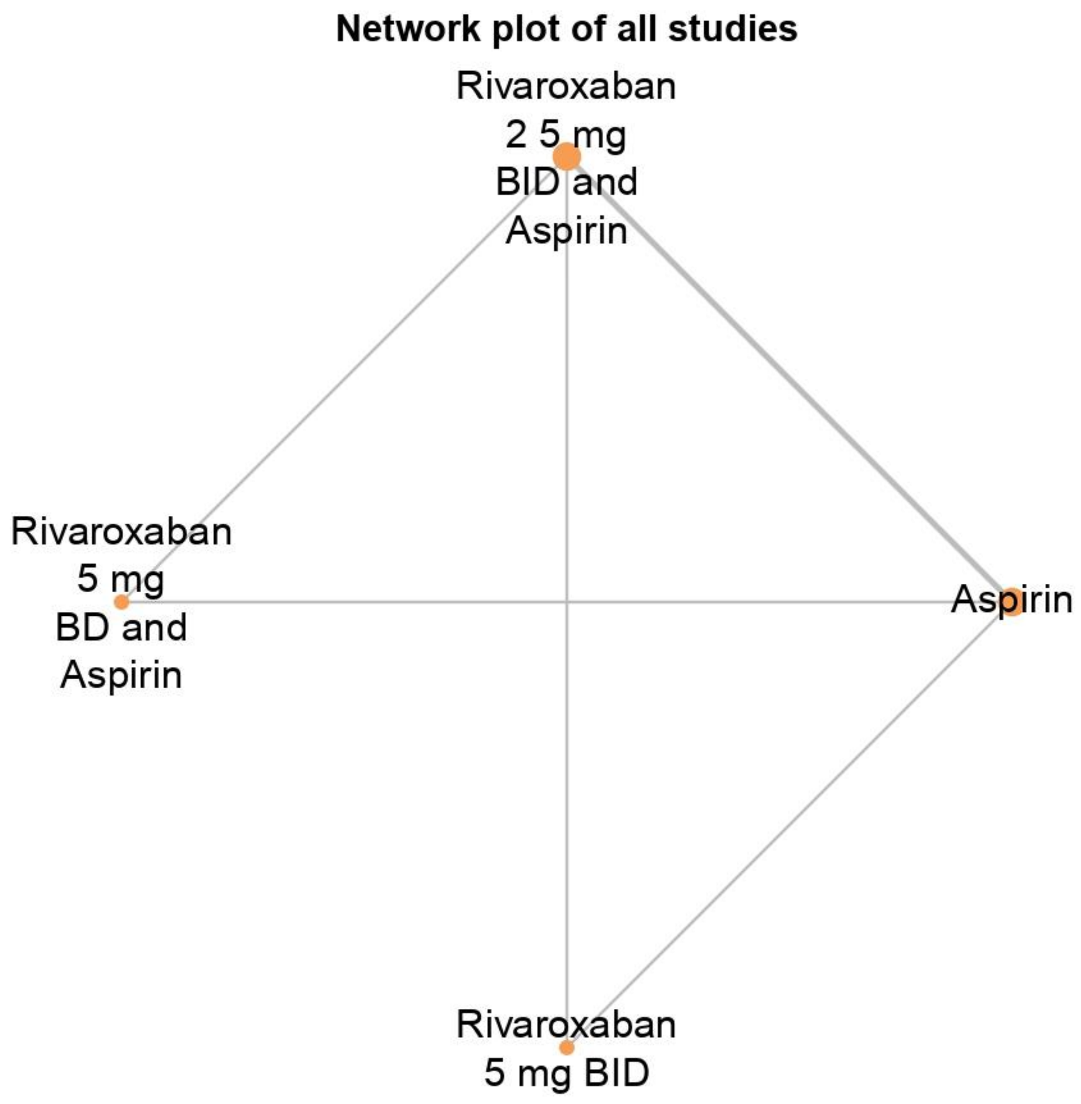

Fig.S7.: Ischemic stroke (CAD studies)

Rivaroxaban_2_5_mg_BID_and_Aspirin			
0.84 [0.43; 1.63]	Rivaroxaban_5_mg_BID		
0.73 [0.35; 1.53]	0.87 [0.35; 2.16]	Rivaroxaban_5_mg_BD_and_Aspirin	
0.65 [0.38; 1.10]	0.77 [0.40; 1.48]	0.89 [0.43; 1.84]	Aspirin

Table S7.: Ischemic stroke (CAD studies)

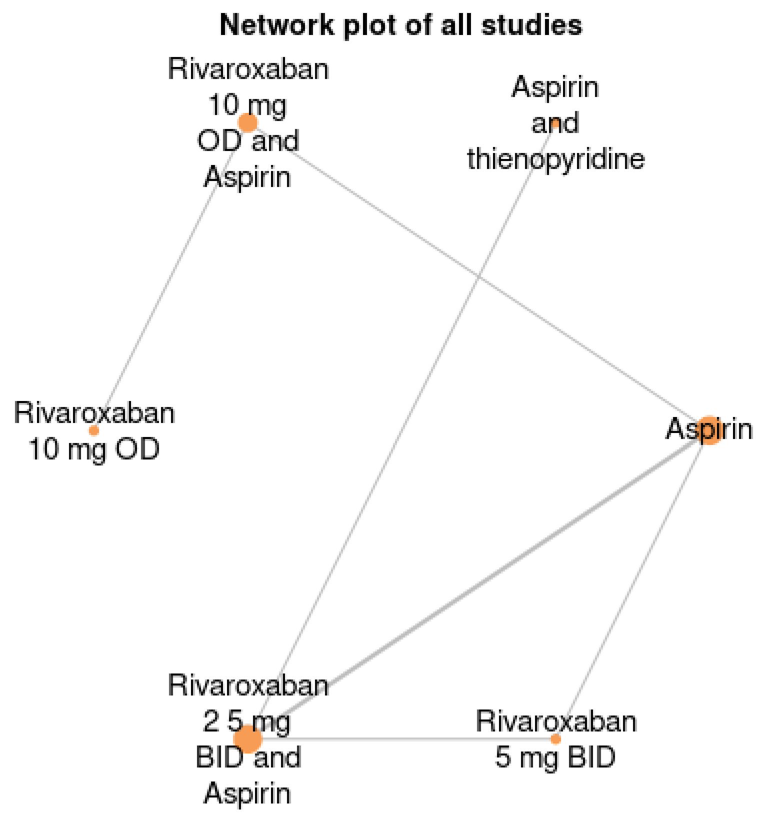

Fig.S8.: Major ISTH bleeding (all included studies)

Aspirin_and_thienopyridine					
0.96 [0.60; 1.55]	Aspirin				
0.99 [0.43; 2.27]	1.03 [0.52; 2.03]	Rivaroxaban_10_mg_OD			
0.68 [0.41; 1.12]	0.71 [0.52; 0.95]	0.69 [0.33; 1.45]	Rivaroxaban_5_mg_BID		
0.60 [0.30; 1.18]	0.62 [0.38; 1.02]	0.60 [0.38; 0.96]	0.88 [0.49; 1.57]	Rivaroxaban_10_mg_OD_and_Aspirin	
0.61 [0.40; 0.92]	0.63 [0.50; 0.80]	0.62 [0.30; 1.27]	0.89 [0.67; 1.19]	1.02 [0.59; 1.77]	Rivaroxaban_2_5_mg_BID_and_Aspirin

Table S8.: Major ISTH bleeding (all included studies)

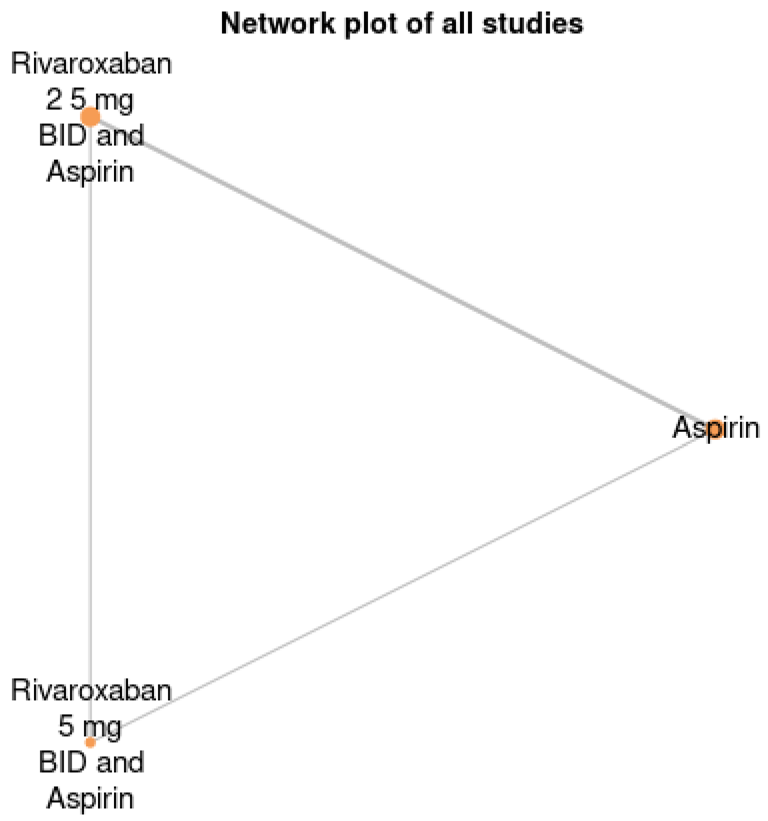

Fig.S9.: Timi major bleeding (all included studies)

Aspirin		
0.70 [0.13; 3.72]	Rivaroxaban_2_5_mg_BID_and_Aspirin	
0.36 [0.04; 3.33]	0.52 [0.06; 4.71]	Rivaroxaban_5_mg_BID_and_Aspirin

Table S9.: Timi major bleeding (all included studies)

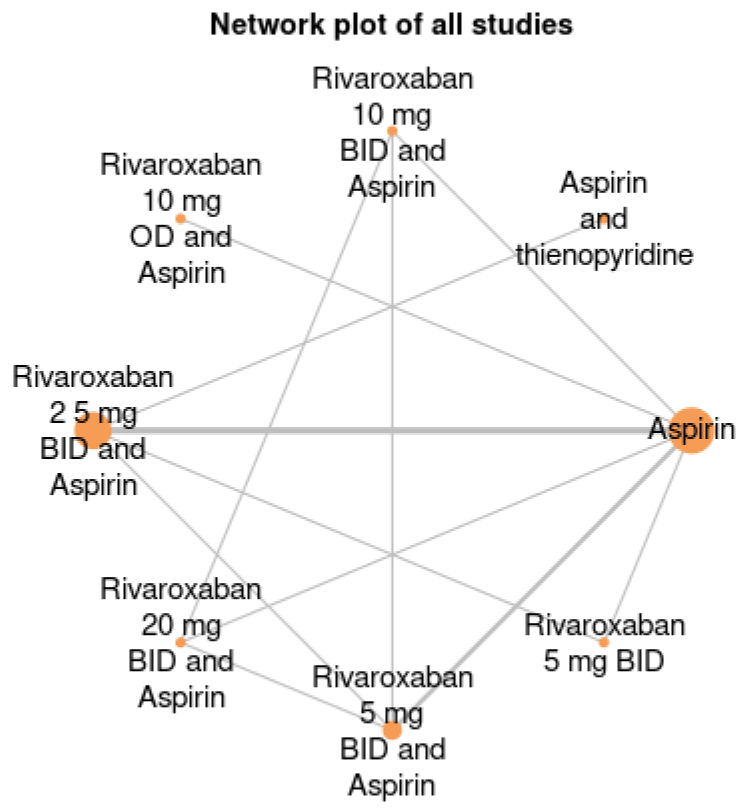

Fig.S10.: Fatal bleeding (all included studies)

Aspirin							
0.95 [0.55; 1.64]	Rivaroxaban_2_5_mg_BID_and_Aspirin						
0.95 [0.33; 2.78]	1.00 [0.40; 2.52]	Aspirin_and_thienopyridine					
0.88 [0.42; 1.83]	0.93 [0.47; 1.85]	0.93 [0.29; 2.93]	Rivaroxaban_5_mg_BID				
0.50 [0.05; 5.56]	0.53 [0.05; 6.23]	0.53 [0.04; 7.34]	0.57 [0.05; 7.04]	Rivaroxaban_10_mg_OD_and_Aspirin			
0.55 [0.28; 1.07]	0.58 [0.28; 1.21]	0.58 [0.18; 1.88]	0.62 [0.25; 1.58]	1.09 [0.09; 13.08]	Rivaroxaban_5_mg_BID_and_Aspirin		
0.33 [0.12; 0.92]	0.34 [0.11; 1.08]	0.34 [0.08; 1.49]	0.37 [0.11; 1.30]	0.65 [0.05; 8.83]	0.60 [0.20; 1.74]	Rivaroxaban_10_mg_BID_and_Aspirin	
0.14 [0.06; 0.35]	0.15 [0.05; 0.42]	0.15 [0.04; 0.59]	0.16 [0.05; 0.51]	0.28 [0.02; 3.63]	0.26 [0.10; 0.67]	0.43 [0.19; 0.99]	Rivaroxaban_20_mg_BID_and_Aspirin

Table S10.: Fatal bleeding (all included studies)

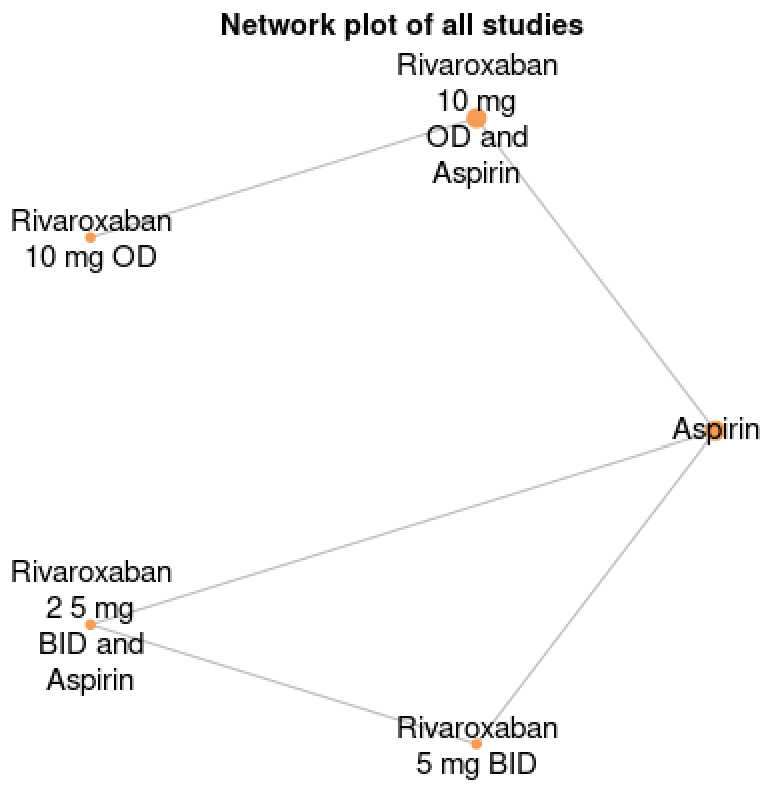

Fig.S11.: Hemorrhagic stroke (all included studies)

Rivaroxaban_10_mg_OD		.		
0.31 [0.10; 0.94]	Rivaroxaban_10_mg_OD_and_Aspirin			
0.20 [0.02; 1.67]	0.66 [0.11; 3.94]	Aspirin		
0.14 [0.01; 1.29]	0.44 [0.06; 3.13]	0.67 [0.30; 1.49]	Rivaroxaban_2_5_mg_BID_and_Aspirin	
0.08 [0.01; 0.70]	0.24 [0.04; 1.68]	0.37 [0.18; 0.76]	0.55 [0.29; 1.04]	Rivaroxaban_5_mg_BID

Table S11.: Hemorrhagic stroke (all included studies)

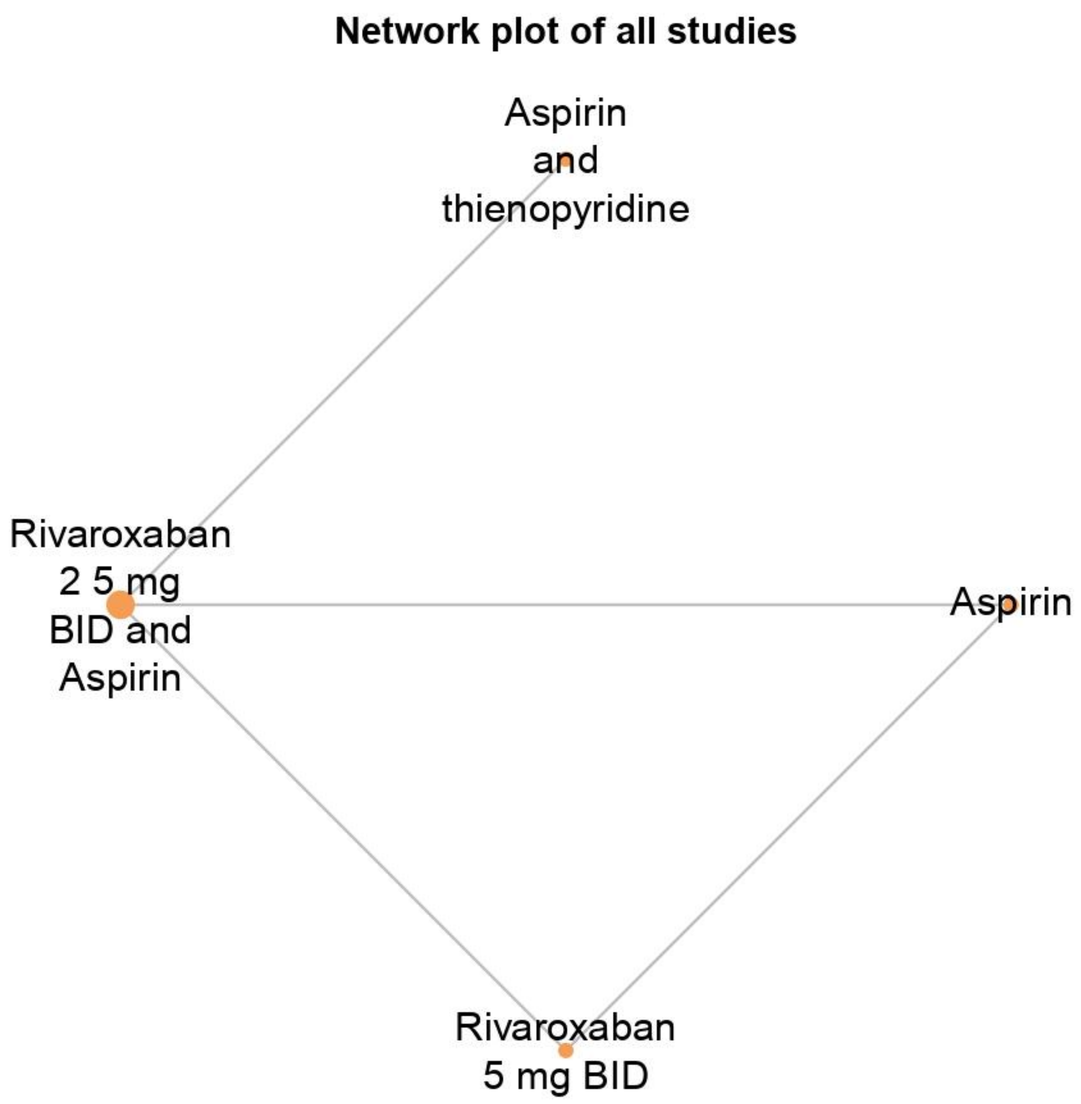

Fig.S12.: Major ISTH bleeding (CAD studies)

Aspirin			
0.93 [0.61; 1.41]	Aspirin_and_thienopyridine		
0.66 [0.52; 0.84]	0.71 [0.48; 1.06]	Rivaroxaban_5_mg_BID	
0.56 [0.45; 0.71]	0.61 [0.43; 0.86]	0.85 [0.70; 1.04]	Rivaroxaban_2_5_mg_BID_and_Aspirin

Table S12.: Major ISTH bleeding (CAD studies)

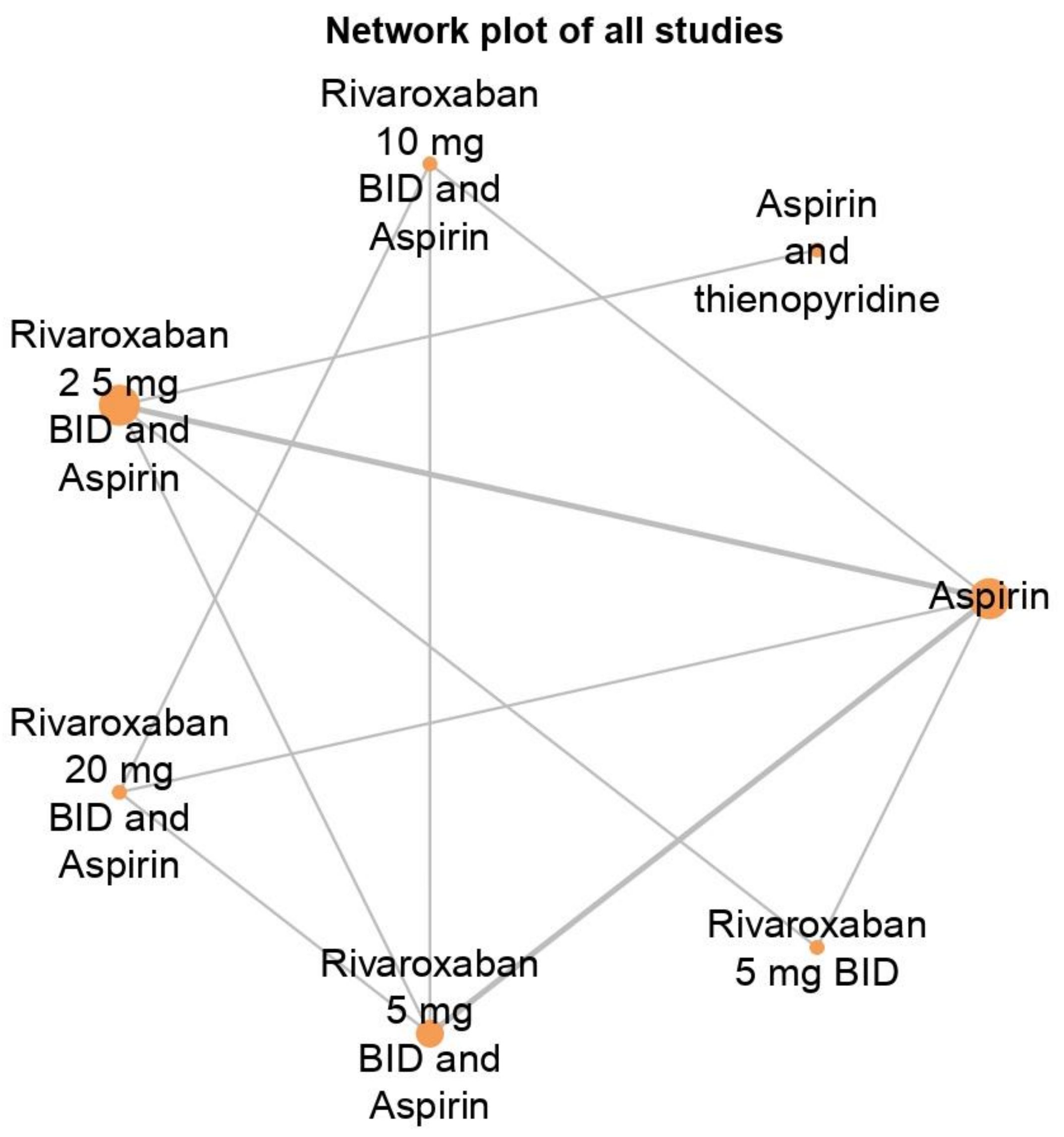

Fig.S13.: Fatal bleeding (CAD studies)

Aspirin						
0.93 [0.50; 1.74]	Rivaroxaban_2_5_mg_BID_and_Aspirin					
0.94 [0.31; 2.85]	1.00 [0.40; 2.52]	Aspirin_and_thienopyridine				
0.87 [0.41; 1.85]	0.93 [0.46; 1.88]	0.93 [0.29; 2.96]	Rivaroxaban_5_mg_BID			
0.54 [0.28; 1.07]	0.58 [0.27; 1.26]	0.58 [0.18; 1.94]	0.63 [0.25; 1.59]	Rivaroxaban_5_mg_BID_and_Aspirin		
0.33 [0.11; 0.92]	0.35 [0.11; 1.12]	0.35 [0.08; 1.54]	0.37 [0.11; 1.32]	0.60 [0.20; 1.74]	Rivaroxaban_10_mg_BID_and_Aspirin	
0.14 [0.06; 0.35]	0.15 [0.05; 0.44]	0.15 [0.04; 0.61]	0.16 [0.05; 0.51]	0.26 [0.10; 0.67]	0.43 [0.19; 0.99]	Rivaroxaban_20_mg_BID_and_Aspirin

Table S13.: Fatal bleeding (CAD studies)

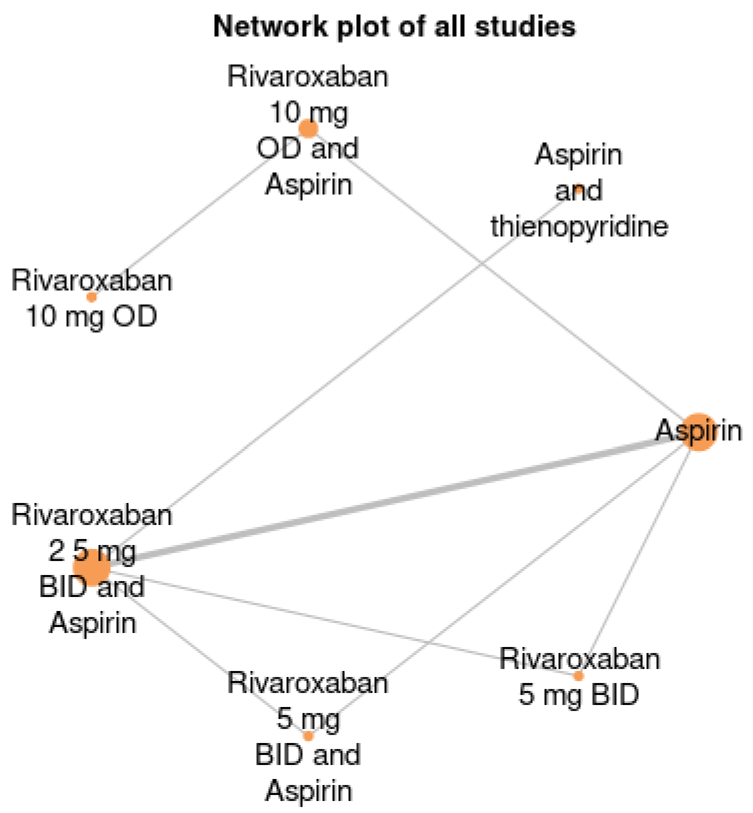

Fig.S14.: Death from any cause (all included studies)

Rivaroxaban_2_5_mg_BID_and_Aspirin						
0.95 [0.64; 1.43]	Aspirin_and_thienopyridine					
0.91 [0.41; 2.06]	0.96 [0.39; 2.37]	Rivaroxaban_10_mg_OD				
0.87 [0.59; 1.27]	0.91 [0.52; 1.59]	0.95 [0.40; 2.25]	Rivaroxaban_5_mg_BID	0.97 [0.64; 1.47]		
0.86 [0.67; 1.10]	0.90 [0.56; 1.44]	0.94 [0.43; 2.03]	0.99 [0.67; 1.45]	Aspirin		
0.82 [0.54; 1.25]	0.86 [0.48; 1.54]	0.90 [0.37; 2.17]	0.95 [0.56; 1.62]	0.96 [0.64; 1.46]	Rivaroxaban_5_mg_BID_and_Aspirin	
0.51 [0.28; 0.94]	0.54 [0.26; 1.11]	0.56 [0.33; 0.96]	0.59 [0.30; 1.16]	0.60 [0.34; 1.04]	0.62 [0.31; 1.24]	Rivaroxaban_10_mg_OD_and_Aspirin

Table S14.: Death from any cause (all included studies)

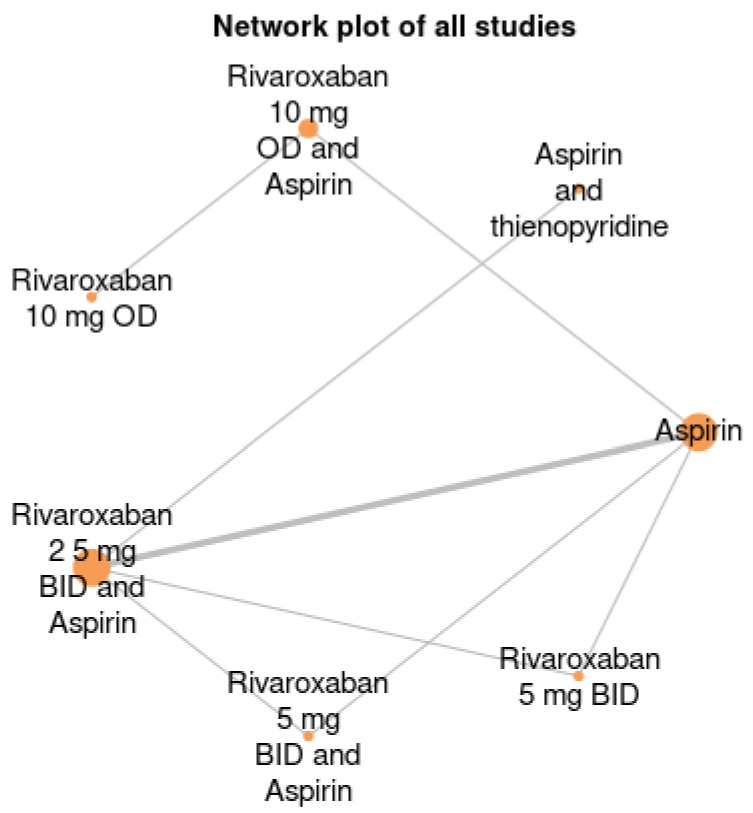

Fig.S15.: Death from CVS cause (all included studies)

Rivaroxaban_2_5_mg_BID_and_Aspirin						
1.09 [0.38; 3.09]	Rivaroxaban_10_mg_OD					
0.95 [0.57; 1.60]	0.88 [0.27; 2.81]	Aspirin_and_thienopyridine				
0.85 [0.52; 1.39]	0.78 [0.26; 2.36]	0.89 [0.43; 1.82]	Rivaroxaban_5_mg_BID			
0.84 [0.61; 1.16]	0.78 [0.29; 2.09]	0.88 [0.48; 1.63]	1.00 [0.61; 1.64]	Aspirin		
0.81 [0.49; 1.36]	0.75 [0.24; 2.27]	0.85 [0.41; 1.76]	0.96 [0.49; 1.88]	0.96 [0.58; 1.60]	Rivaroxaban_5_mg_BID_and_Aspirin	
0.66 [0.30; 1.43]	0.60 [0.30; 1.21]	0.69 [0.27; 1.75]	0.78 [0.33; 1.84]	0.78 [0.38; 1.58]	0.81 [0.34; 1.94]	Rivaroxaban_10_mg_OD_and_Aspirin

Table S15.: Death from CVS cause (all included studies)

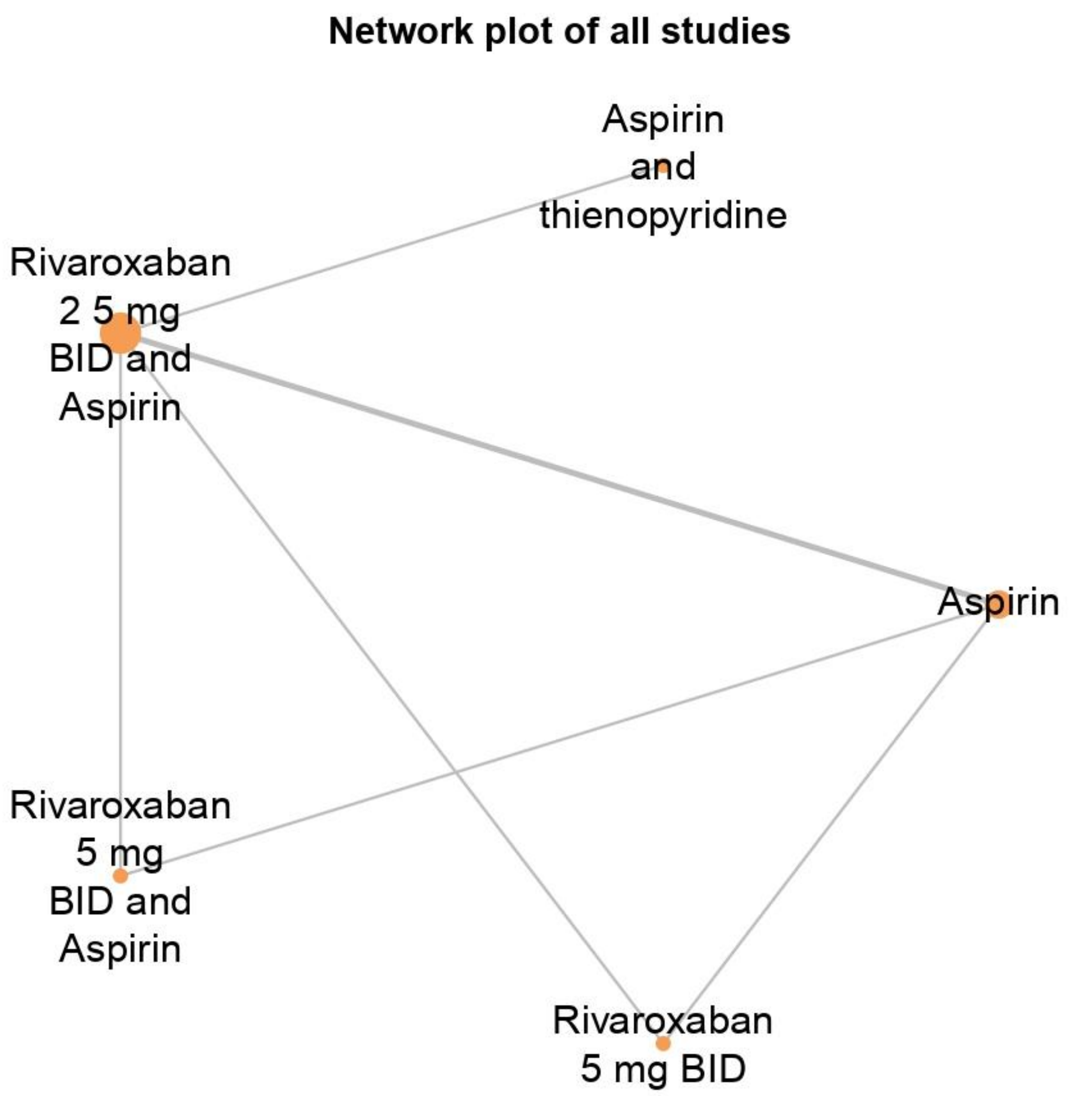

Fig.S16.: Death from any cause (CAD studies)

Rivaroxaban_2_5_mg_BID_and_Aspirin				
0.95 [0.77; 1.19]	Aspirin_and_thienopyridine			
0.82 [0.65; 1.03]	0.86 [0.62; 1.18]	Rivaroxaban_5_mg_BID		
0.78 [0.58; 1.04]	0.82 [0.57; 1.17]	0.95 [0.68; 1.34]	Rivaroxaban_5_mg_BID_and_Aspirin	
0.76 [0.63; 0.93]	0.80 [0.60; 1.07]	0.93 [0.74; 1.18]	0.98 [0.74; 1.30]	Aspirin

Table S16.: Death from any cause (CAD studies)

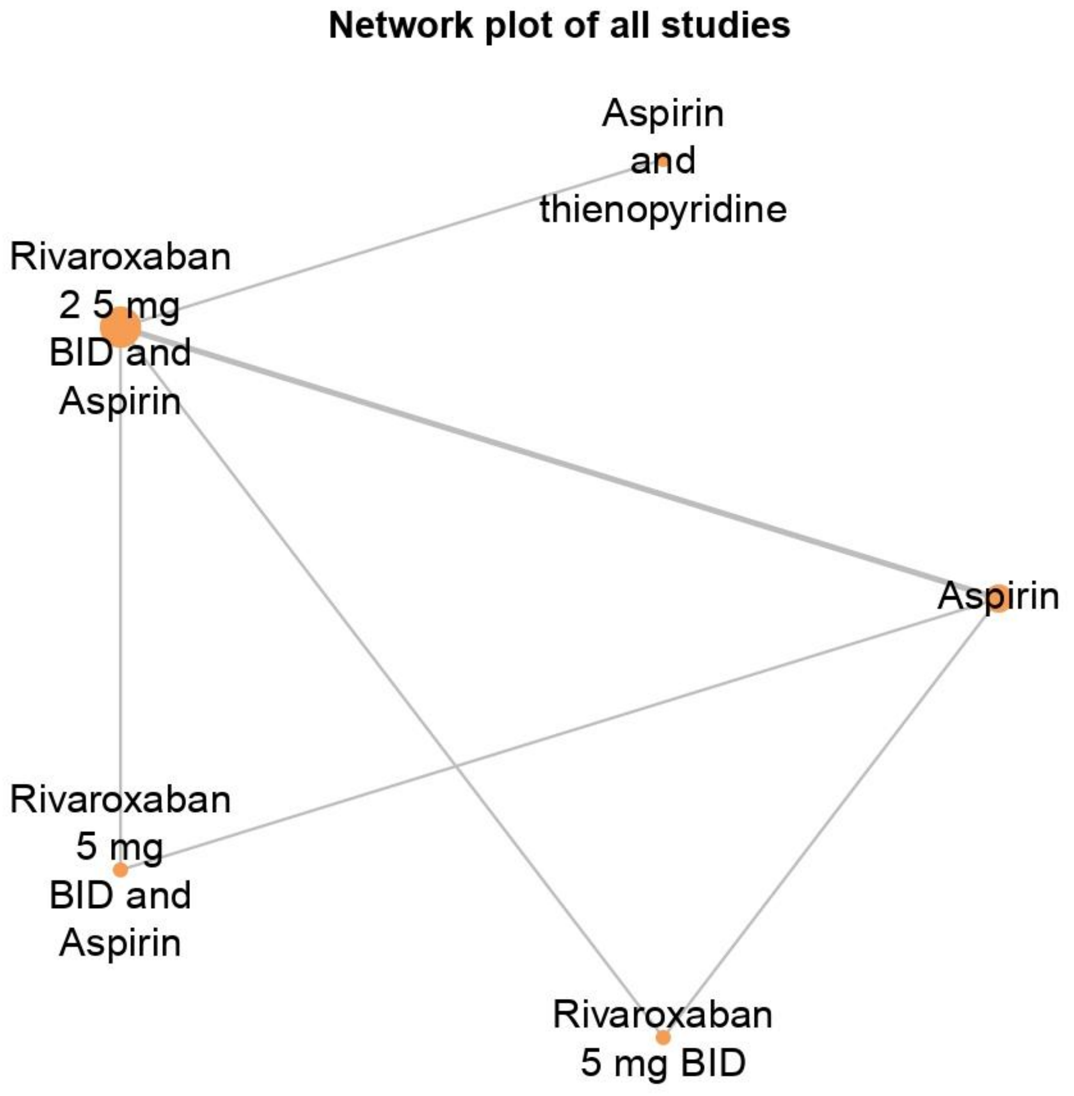

Fig.S17.: Death from CVS cause (CAD studies)

Rivaroxaban_2_5_mg_BID_and_Aspirin				
0.95 [0.83; 1.10]	Aspirin_and_thienopyridine			
0.79 [0.64; 0.97]	0.82 [0.64; 1.06]	Rivaroxaban_5_mg_BID		
0.76 [0.59; 0.97]	0.80 [0.60; 1.06]	0.97 [0.72; 1.29]	Rivaroxaban_5_mg_BID_and_Aspirin	
0.73 [0.62; 0.87]	0.77 [0.61; 0.96]	0.93 [0.76; 1.14]	0.96 [0.76; 1.22]	Aspirin

Table S17.: Death from CVS cause (CAD studies)
